# Wireless In Situ Catalytic Electron Signaling‐Mediated Transcriptomic Reprogramming for Neuron Regeneration via Adaptable Antennas

**DOI:** 10.1002/advs.202504786

**Published:** 2025-05-11

**Authors:** Hoi Man Iao, Chih‐Ying Chen, Ya‐Hui Lin, Wan‐Chi Pan, Chun‐Yi Liang, Hsiu‐Ching Liu, Lo‐Jei Ching, Pei‐Yu Weng, Min‐Ren Chiang, Ru‐Siou Hsu, Tsu‐Chin Chou, I‐Chi Lee, Lun‐De Liao, Li‐An Chu, Shih‐Hwa Chiou, Shang‐Hsiu Hu

**Affiliations:** ^1^ Department of Biomedical Engineering and Environmental Sciences National Tsing Hua University Hsinchu 300044 Taiwan; ^2^ Department of Medical Research Taipei Veterans General Hospital Taipei 112201 Taiwan; ^3^ Institute of Pharmacology College of Medicine National Yang Ming Chiao Tung University Taipei 112304 Taiwan; ^4^ Brain Research Center National Tsing Hua University Hsinchu 300044 Taiwan; ^5^ Department of Biomedical Engineering National Yang Ming Chiao Tung University Taipei 112304 Taiwan; ^6^ Institute of Analytical and Environmental Sciences National Tsing Hua University Hsinchu 300044 Taiwan; ^7^ Institute of Biomedical Engineering and Nanomedicine National Health Research Institutes Miaoli County 35053 Taiwan

**Keywords:** adaptable microbeads, catalytic effect, electron signaling, transcriptomic reprogramming, nerve regeneration

## Abstract

Electron signaling and oxygen level are vital for regulating neural‐cell fate and brain recovery. However, clinical challenges arise from the short half‐life and the difficulty of spatiotemporally controlled oxygen release and electric signals. In this study, a wireless‐charging sustained oxygen release from conductive microgels (SOCO) served as an antenna and an on‐demand O_2_ release for nerve regeneration is developed. Introducing “electromagnetic messenger”, using external alternating magnetic field (AMF) to enhance catalytic oxygen release and electrical stimulation to promote the reconstruction of blood vessels and neurons in vivo. SOCO also reduces TBI glial scarring by reducing activated microglia and stellate cells, promoting infiltration of new neurons. In whole‐brain analyses, effective somatostatin (Sst) production inhibits gamma‐aminobutyric acid (GABA) synthesis in injured areas, thereby improving brain function and behavioral recovery. Furthermore, spatial multiomics combined with single‐cell deconvolution analysis reveals the treatment reprogramming in vivo brain transcriptome of angiogenic markers (Il1a, Lgals3) and GABAergic pathway via modulation of GAD65/67 activity, guiding angiogenesis and neuronal regeneration. This in situ catalytic SOCO with noncontact AMF presents an “electromagnetic messenger”‐based therapeutics for reprogramming the neuro‐regeneration and brain function recovery in TBI.

## Graphical abstract

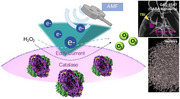

## 1 Introduction

Traumatic brain injury (TBI) is a leading cause of death and long‐term disability in industrialized countries.^[^ ^1^ ^]^ The global cost of TBI is estimated to be as high as $400 billion due to its lasting effects on mortality, cognitive performance, and morbidity.^[^ ^2^ ^]^ There are currently no effective strategies to promote nerve regeneration or functional recovery in TBI.^[^ ^3^ ^]^ After injury, activated stellate cells and infiltrating microglia exacerbate inflammation, leading to glial scarring and neuronal death in the area surrounding the trauma.^[^ ^3^, ^4^, ^5^, ^6^ ^]^ This inflammatory response impedes angiogenesis and neurogenesis, leading to cerebral atrophy, or shrinkage of the brain. Despite recent progress in developing the complex pathophysiology of TBI, the difficulties in nerve regeneration still stems from the heterogeneity and high complexity of TBI.^[^ ^7^ ^]^ For example, small‐molecule drugs are easy to administer but often suffer from poor target specificity and rapid clearance, limiting their effectiveness in the nerve repair.^[^ ^8^ ^]^ Growth factors like nerve growth factor (NGF) and brain‐derived neurotrophic factor (BDNF) face challenges in maintaining therapeutic levels due to short half‐lives and diffusion limits;^[^ ^9^ ^]^ and nucleic acid delivery, including gene therapy, is hindered by inefficient transfection, and off‐target effects, restricting clinical translation.^[^ ^10^ ^]^

To address this issue, one possible solution is to enhance electron signaling of cells to program neuron function.^[^ ^11^, ^12^, ^13^ ^]^ This bioelectrical interconnection circuit acts as an endogenous electrical current, allowing neuron communication and regeneration. In clinics, non‐invasive neuromodulation techniques, including deep brain stimulation (DBS), transcranial direct current stimulation (tDCS), and transcranial magnetic stimulation (TMS), have been employed to treat various brain disorders by controlling brain circuit dynamics.^[^ ^14^, ^15^ ^]^ The electromagnetic fields activate electron signaling between neurons and promotes neuronal differentiation through Ca^2+^ signaling pathways.^[^ ^16^ ^]^ In this regard, non‐invasive electrical currents generated by specific nanoconductors (such as gold and graphene oxide sheets) upon exposure to alternating magnetic fields (AMF) can promote neuron activation.^[^ ^17^ ^]^ Although biowireless electronic signaling is still in its early stages, it shows great potential in promoting neuron communications and neurogenesis in neurodegenerative diseases such as Parkinson's disease.^[^ ^18^, ^19^, ^20^, ^21^, ^22^, ^23^, ^24^, ^25^ ^]^ This emphasizes the need for technologies that can electrically interact with nerve cells, whose function is intrinsically linked to electron transfer.^[^ ^26^, ^27^ ^]^

Maintaining optimal oxygen levels is critical for creating a favorable environment that supports neurogenesis.^[^ ^28^, ^29^, ^30^ ^]^ It promotes neuronal proliferation, differentiation, and survival, all of which are essential for effective neurogenesis. Hypoxia or fluctuating oxygen levels can impair these processes, while sustained oxygenation enhances the microenvironment necessary for neural stem cell activity and the formation of new neurons. Recent studies have highlighted its critical involvement in reducing inflammation and promoting neovascularization.^[^ ^31^, ^32^, ^33^ ^]^ Several oxygen delivery platforms, such as hyperbaric oxygen therapy (HBOT), oxygen carriers (e.g., perfluorocarbons and hemoglobin‐based vehicles), and oxygen‐generating biomaterials (e.g., catalase‐peroxide platforms), have been developed to enhance oxygen availability.^[^ ^34^, ^35^, ^36^ ^]^ However, these techniques face significant challenges, including the pulmonary toxicity associated with HBOT, reducing oxygen diffusion and capacity.^[^ ^37^, ^38^, ^39^ ^]^

**Figure 1:**
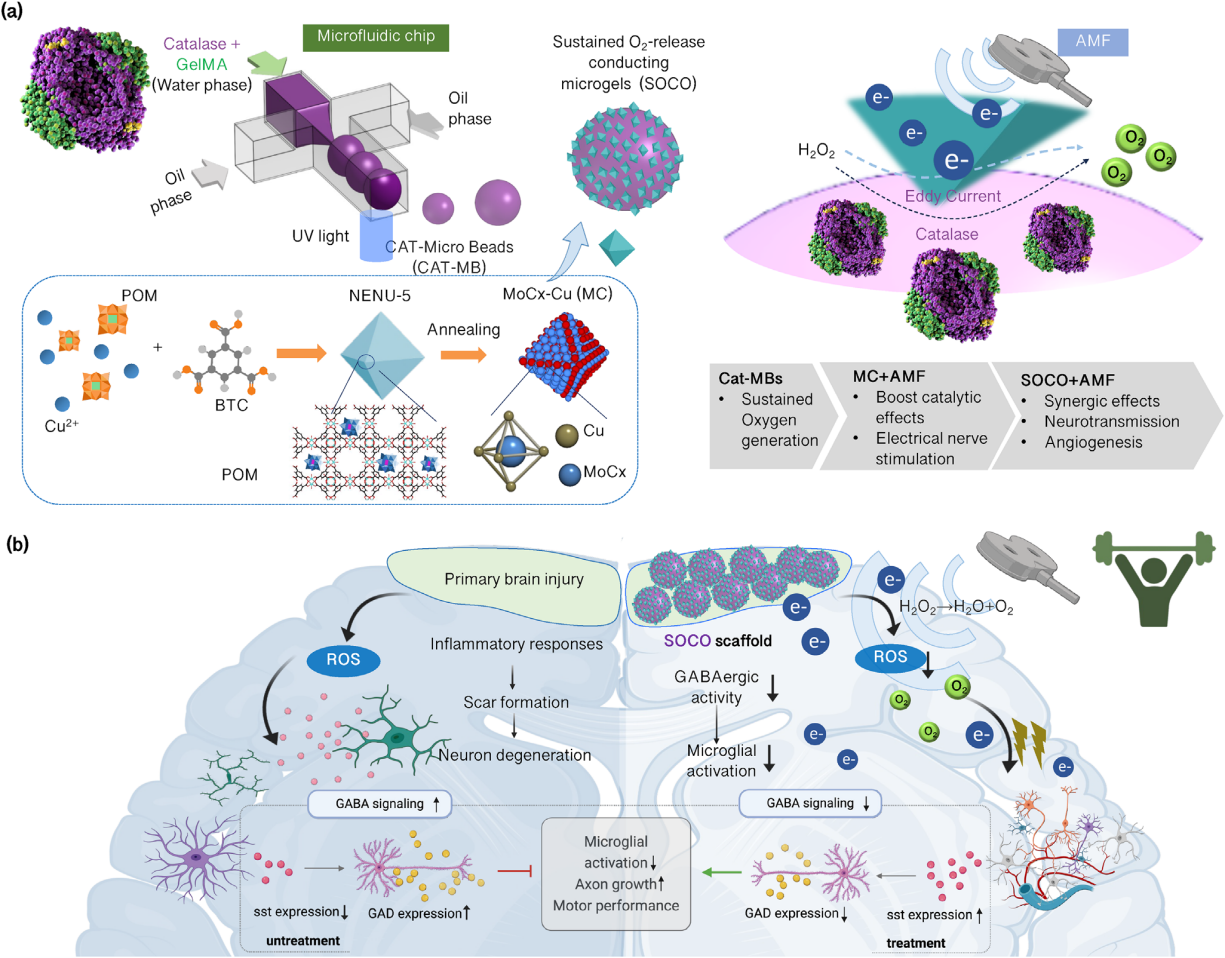
The alternating magnetic field (AMF)‐responsive electric stimulus of sustained O_2_‐release conducting microgels (SOCO) is an innovative device designed to selectively activate treatments for traumatic brain injury (TBI). This system leverages catalase‐encapsulated microgels to facilitate controlled oxygen release in response to AMF, enabling precise and targeted therapeutic interventions for TBI. a) The scheme illustrated the SOCO preparation, which involves catalase encapsulating in microbeads (CAT‐MB) with a conducting MC. b) SOCO facilitates targeted delivery for oxygen release and electron delivery at TBI treatments, leading to angiogenesis, neurogenesis, and functional recovery.

Here, inspired by electron signaling and oxygen level in nerve regeneration, we developed a sustained oxygen‐release conducting microgel (SOCO) coated by a porous molybdenum carbide octahedron (MC) featuring catalytic oxygen release and a wirelessly electric signaling for programming the never regeneration in TBI (**Figure** **1**a). Selective activation of SOCO exhibited the reduction of the activated microglia and astrocytes, mitigating the formation of glial scaring. Subsequently, the synergistic treatments of electric signaling and catalytic‐boosting oxygen release promote angiogenesis, neurogenesis, and functional recovery in vivo (Figure 1b). Furthermore, this process also increases the content of whole‐brain somatostatin (Sst), promoting the GABAergic pathways and the recovery of brain function.

In TBI, spatiotemporal transcriptomics (ST) has become a powerful tool for unraveling the intricate cellular responses to injury and the dynamic changes in gene expression.^[^ ^40^, ^41^, ^42^ ^]^ After treatment, ST combined with single‐cell deconvolution analysis demonstrated modulation of microglial activation and supported neuronal regeneration through transcriptome reprogramming. Angiogenic growth markers, such as ***Il1a, Lgals3, Anxa3***, and ***Cybb***, were elevated in SOCO+AMF‐treated sample. This in situ catalytic microgel combined with noncontact AMF therapy provides a promising treatment strategy for TBI patients.

Compared to our previous study,^[^ ^23^, ^24^ ^]^ this is the first study to use an alternating magnetic field (AMF) to locally enhance catalase‐driven catalytic oxygen production (an external stimulus in enzyme‐based catalysis). It integrates electrical stimulation with transcriptomic reprogramming to promote neuron regeneration and facilitate the reconstruction of blood vessels and neurons in vivo. Through the observation, this study's novelty shines through its revolutionary approach, distinctly targeting activated microglia and stellate cells to reduce glial scarring—a mechanism absent in previous study—while employing spatial multiomics and single‐cell deconvolution to drive in vivo reprogramming of angiogenic markers (e.g., Il1a, Lgals3) and GABAergic pathways (via GAD65/67); furthermore, it pioneers the enhancement of somatostatin production to inhibit GABA synthesis.

## 2 Results and Discussion

### 2.1 Synthesis and Characterization of NENU‐5 and MC (MC)

**Figure 2:**
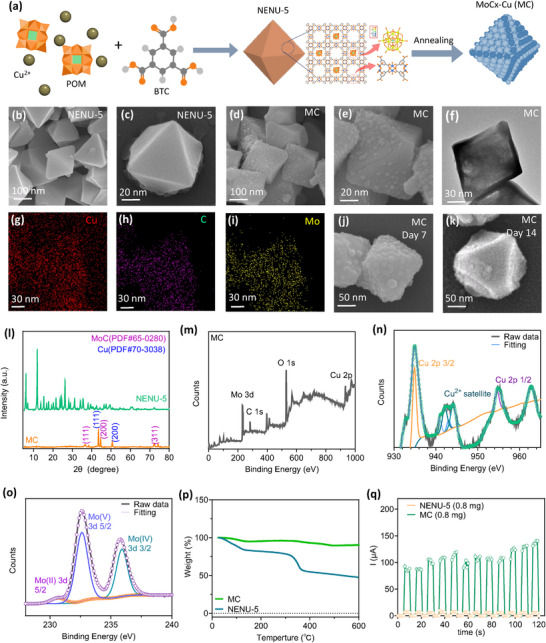
Preparation and characterization of MoCx‐Cu (MC). a) Schematic illustration of the preparation of MC. b–e) SEM images of NENU‐5 and MC. f–i) TEM image and element mapping of MC. j–k) SEM images of NENU‐5 and MC after 14 d. l) X‐ray diffraction patterns of NENU‐5 and MC. m–o) XPS spectra of MC. p) TGA analysis of MC and NENU‐5. q) *I*–*T* curves of NENU‐5 and MC.

The SOCO was synthesized based on a MOF‐assisted method, involving the formation of porous molybdenum carbide octahedral nanoparticles (MC, annealed NENU‐5) in **Figure** **2**a. The process was driven by confined carburization process between MOF's organic ligands and polyoxometalates (POMs) within pores, which enabled the transition of metal carbides into MOF's structure. The organic ligands ensured an even distribution of the POMs at the atomic level, guiding a uniform carburization reaction. During annealing, the precursor [Cu_2_(BTC)_4_/3(H_2_O)_2_]6[H_3_PMo_12_O_40_] (NENU‐5; BTC = benzene‐1,3,5‐tricarboxylate) facilitated the interaction between Mo‐based Keggin‐type POMs (H_3_PMo_12_O_40_) and carbonaceous species in the BTC ligands, leading to the formation of MC.

Scanning electron microscopy (SEM) images showed that both NENU‐5 and MC particles exhibited octahedral morphologies with side lengths of ≈180 nm (Figure 2b–e). The surface of MC appeared rougher compared to NENU‐5, likely due to the effects of the annealing process. This roughness was further confirmed by transmission electron microscopy (TEM) of MC, which also showed a textured surface (Figure 2f). Energy dispersive spectroscopy (EDS) mapping exhibited several elements within MC including Cu, C, and Mo (Figure 2g–i). The annealing process contributed to reducing dislocations and promoting atom migration within the lattice, leading to the elimination of displacements, crystal form rearrangement, and retention of key elements such as Cu and Mo. The surface of MC showed slight dissolution after 7 and 14 d (Figure 2j,k).

X‐ray diffraction (XRD) patterns for NENU‐5 and MC were illustrated in Figure 2l. Prior to annealing, the NENU‐5 showed low crystallinity, indicating a relatively disordered atomic structure. After annealing, the MC displayed distinct peaks in its XRD pattern, corresponding to the characteristic peaks of MoO_2_ (JCPDS 32–0671) and Cu (JCPDS 04–0836). It suggests the formation of copper oxide (CuO) and copper(I) oxide (Cu_2_O) within the material. The heat treatment process facilitated the recrystallization of elements in NENU‐5, resulting in the formation of various crystalline structures within the MC composite, which in turn contributes to the material's distinctive properties and potential applications.

X‐ray photoelectron spectroscopy (XPS) was utilized to analyze the surface of elemental and atomic compositions. The XPS results confirmed that the elements including Mo, Cu, P, O, and C presented in both NENU‐5 and MC (Figure 2m–o; Figure S1 in the Supporting Information). Base on the spectra of NENU‐5 and MC, the binding energies for Mo 3d peaks at 233.3 and 231.5 eV corresponded to Mo(IV) 3d3/2 and Mo(V) 3d5/2, respectively. A distinctive peak at 943 eV only shown in MC indicated a strong Cu2+ satellite. The thermal stability of MC could be observed based on the thermogravimetric analysis (TGA) analysis (Figure 2p). Furthermore, the induced currents generated by MC was greater than the current produced by NENU‐5 under AMF, attributed to MC's enhanced crystallinity and conductivity (Figure 2q). The electromagnetic response of materials was measured using a fluorine‐doped tin oxide (FTO) electrode by coating the materials onto the substrate. When the materials contain MB, some MC may not directly contact the electrode, leading to a weaker electromagnetic response. The Brunauer–Emmett–Teller (BET) method was employed to determine the surface area of particles (Figure S2; Supporting Information). Moreover, MC exhibited hysteresis loops in their surface areas. Additionally, the pore size of MC was smaller than that of NENU‐5 (Figure S2; Supporting Information), likely due to atom migration and recrystallization following the annealing process. Compared to gold particles, the MC alloy system is well‐suited to serve as a nanoantenna, offering scalable production and good in vivo stability. The carbonized surface of MC is also well‐suited for adsorbing catalase.

### 2.2 Synthesis and Characterization of MB and SOCO

**Figure 3:**
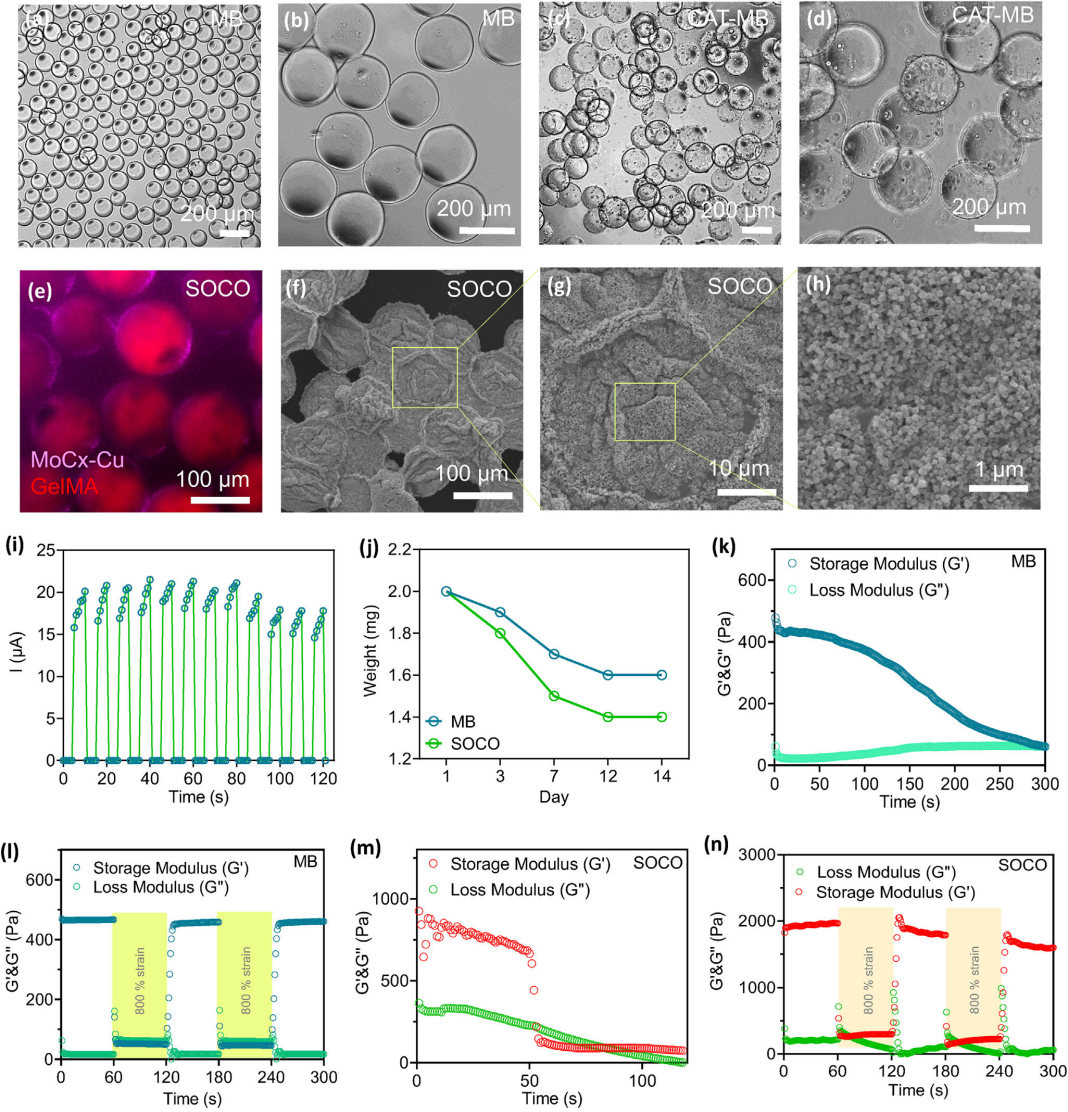
Preparation and characterization of MB, CAT‐MB, and SOCO. a–d) Microscope images of MB and CAT‐MB. e) CLSM image of SOCO. f–h) SEM images of SOCO. i) *I*–*T* curve of SOCO. j) Degradation of MB and SOCO. k–n) Damage healing properties of MB and SOCO.

Microporous annealed particle (MAP) scaffolds are an innovative particulate material composed of tunable microgels that form a network of interconnected void spaces.^[^ ^43^ ^]^ To produce MBs, gelatin methacryloyl (GelMA) was first synthesized by conjugating methacrylamide to gelatin. Using a microfluidic flow‐focusing chip, GelMA MBs were generated in high throughput with uniform size distribution (**Figure** **3**a,b; Figure S3, Supporting Information). After photocrosslinking, the GelMA droplets exhibited a narrow size distribution. The MBs were washed to remove oil and then suspended into the water, where the crosslinked MBs displayed a larger average diameter of 180 µm. Upon loading with catalase (CAT), the surface of the MBs became rougher while maintaining their overall morphology (Figure 3c,d).

To coat MC onto CAT‐MB, a straightforward process is used: MC is directly added to the CAT‐MB solution. The strong electrostatic interactions between the negatively charged MB and the positively charged MC drive the assembly of these components. This electrostatic attraction facilitates the close binding of MC to the surface of CAT‐MB, resulting in the formation of the SOCO. Notably, the strong electrostatic forces ensure that MC remains uniformly coated on CAT‐MB, contributing to the stability and functionality of the resulting SOCO structure. To assess the coating results, both fluorescence imaging and scanning electron microscopy (SEM) were utilized. The fluorescence image in Figure 3e demonstrates that MC was uniformly coated around the surface of MB. SEM analysis provided a detailed view of the SOCO morphology, revealing a high density of MC on the SOCO structure (Figure 3f–h). Collectively, our findings indicate that the coating process was successful, resulting in a thorough and consistent coverage of MC on the surface of MB. The zeta potential results further indicate interactions between MB and MC (Figure S3c; Supporting Information).

The *I*–*T* curve revealed a significant eddy current generated by SOCO under AMF exposure, which can be attributed to the improved crystallinity and conductivity of MC (Figure 3i). This enhanced performance is likely due to the well‐organized crystalline structure of MC, which facilitates more efficient electron flow and, consequently, stronger eddy currents. This combination of enhanced crystallinity and conductivity in MC contributes to the superior electromagnetic properties of SOCO. The degradation of MB and SOCO were also evaluated by weight of materials in PBS at 37 °C. The degradation of MB and SOCO was assessed by monitoring the weight loss of materials immersed in PBS at 37 °C containing 0.1 mg mL^−1^ of collagenase (Figure 3j). The weight loss was primarily attributed to MB. The difference between MB and SOCO may be due to surface degradation, where some MC was lost, while the remaining SOCO was collected using a mesh.

Rheological tests were performed on MB and SOCO to assess their behavior under different shear conditions. For MB, significant deformation was observed under a shear stress of 85.9 Pa at 30 Hz, achieving a shear strain of 100% (Figure 3k). Despite this, the microspheres retained their colloidal properties during alternating shear strains of 1% and 100% (Figure 3l). Similarly, SOCO deformed under a shear stress of 58.9 Pa at 50 Hz, with a shear strain of 47% in Figure 3m. These microspheres also maintained their colloidal properties during alternating shear strains of 1% and 47% (Figure 3n), suggesting resilience and stability under varying shear conditions. The elastic moduli of MB at concentrations of 5, 7.5, and 10 wt% were ≈1.2, 2.6, and 4.5 kPa, respectively (Figure S3d; Supporting Information). For further experiments, 5 wt% GelMA, with an elastic modulus of 1.2 kPa, was selected as it closely matches the modulus of cortical tissue.^[^ ^44^ ^]^

### 2.3 H_2_O_2_‐Driven O_2_ Production Profile of Nanozyme‐Reinforced Hydrogel

Injectable microbeads for nerve therapy remain limited, as most existing hydrogels primarily focus on drug delivery without addressing both hypoxia and reactive oxygen species (ROS) in traumatic brain injury (TBI).^[^ ^45^, ^46^ ^]^ However, recent developments in nanozymes offer a promising approach to modulate oxidative stress and hypoxia in diverse oxidative stress‐related conditions.^[^ ^7^ ^]^ Incorporating nanozymes into injectable systems can create advanced bioactive platforms that address complex tissue‐specific challenges, broadening their biomedical applications. Thus, integrating hydrogels with nanozymes may provide a more targeted and multifunctional approach, enabling ROS decomposition, sustained oxygenation, and the creation of a supportive microenvironment for cell growth and guide nerve stem cell differentiation towards desired lineages.

**Figure 4:**
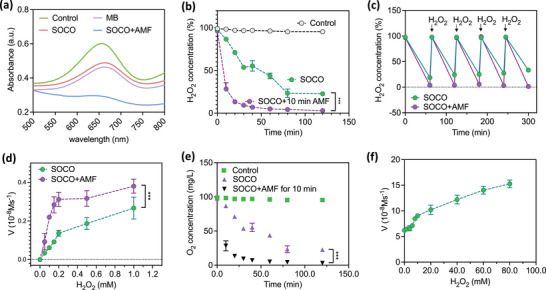
Catalytic activity characterization of CAT, SOCO, and SOCO+AMF. a) UV–vis absorption spectra of CAT, SOCO, and SOCO+AMF with the addition of Ti(SO_4_)_2_ solution. The concentration of H_2_O_2_ was 0.5 × 10^−3^ m. b) Decomposition of H_2_O_2_ (0.5 × 10^−3^ m) with SOCO and SOCO+AMF. (*n* = 5, mean ± s.d., ***p < 0.005, one‐way ANOVA with Tukey's multiple comparison test). c) Repetitive catalytic H₂O₂ consumption ability of SOCO and SOCO+AMF with repeated H₂O₂ additions (0.5 × 10^−3^ m). d) Catalytic kinetics of SOCO and SOCO+AMF with varying H₂O₂ concentrations. The initial reaction velocity (*V*) was monitored in PBS solution (pH 7.0) at 37 °C. (*n* = 5, mean ± s.d., ***p < 0.005, one‐way ANOVA with Tukey's multiple comparison test). e) O₂ generation ability of SOCO and SOCO+AMF with 0.2 × 10^−3^ m H₂O₂. f) Continuous catalytic O₂ generation ability of SOCO with repeated additions of 0.1 × 10^−3^ m H₂O₂.

The exchange of substances during metabolic processes is important to the physiological functions of living organisms. Drawing inspiration from these processes, enzyme‐enhanced self‐protecting SOCO were designed to function as H_2_O_2_‐driven oxygen generators, capable of catalytically breaking down H_2_O_2_ to produce O_2_. The catalase activity within these microbeads was analyzed by measuring H_2_O_2_ consumption using the titanium sulfate [Ti(SO_4_)_2_] colorimetric method. As shown in **Figure** **4**a, the ability of SOCO to decompose H_2_O_2_ was directly related to its catalase content and AMF, indicating that the enzyme was responsible for the superior catalytic performance of SOCO under AMF. To further explore the kinetics of H_2_O_2_ decomposition in the hydrogel, a time‐dependent H_2_O_2_ assay was conducted in the presence of SOCO (Figure 4b).

The addition of H_2_O_2_ (0.5 × 10^−3^ m) to SOCO+AMF resulted in the decomposition of over 75% of the H_2_O_2_ within the first 10 min, with complete breakdown occurring within 120 min. In contrast, no significant reduction in H_2_O_2_ was observed when treated with hydrogel lacking CAT and AMF (control group), confirming that the H_2_O_2_ decomposition was due to the presence of catalase (CAT). Notably, SOCO maintained its catalytic activity over time, continuing to effectively decompose H_2_O_2_ even after repeated additions, without any noticeable delay (Figure 4c). Building on these findings, the hydrogel's ability to generate O_2_ from H_2_O_2_ was further investigated. In Figure 4d, the Michaelis–Menten kinetic parameters for the catalase activity of SOCO were determined using H_2_O_2_ as the substrate. The resulting Michaelis–Menten constant (*K*_m_) and maximum reaction velocity (*V*_max_) values indicated the hydrogel's high catalytic efficiency. Further studies assessed the O_2_ production capabilities of SOCO, revealing that the O_2_ concentration in the solution significantly increased within 20 min of H_2_O_2_ addition, while the hydrogel without the SOCO showed no O_2_ generation (Figure 4e). Moreover, SOCO demonstrated sustained O_2_ production in the presence of H_2_O_2_, highlighting its excellent catalytic stability (Figure 4f).

While it is true that the concentration of extracellular H₂O₂ in injured brain tissue is relatively low, our approach leverages localized and sustained catalysis to maximize therapeutic effects. The encapsulation of CAT within microgel microspheres stabilizes the enzyme and ensures prolonged catalytic activity, allowing continuous oxygen generation even from minimal H₂O₂ sources. Previous studies have demonstrated that enzyme‐based catalytic systems can exert therapeutic effects even under low physiological substrate concentrations.^[^ ^47^ ^]^ Similar biomaterial‐based strategies utilizing peroxidase or catalase have successfully promoted neuroprotection and tissue repair in vivo. Rather than using 0.5 × 10^−3^ m H₂O₂, the concentration was further modified to align with reported physiological levels, which range from 1–100 × 10^−6^ m in pathological conditions such as ischemic stroke and neuroinflammation.^[^ ^48^ ^]^ As shown in Figure S4 in the Supporting Information, the catalytic pattern at 100 × 10^−6^ m H₂O₂ remains comparable to that observed within the physiologically relevant range. This suggests that SOCO retains its catalytic activity even at lower H₂O₂ concentrations at 100 × 10^−6^ m, reinforcing its potential for therapeutic applications in oxidative stress‐related neural disorders.

AFMs enhance the catalytic activity of catalase in the presence of conducting nanoparticles by inducing eddy currents that generate localized minorly heating, improving mass transport, and modulating electron spin states. When AMFs interact with conducting nanoparticles, they produce the surface energy, elevating the kinetic energy near the enzyme's active site and reducing activation barriers for the decomposition of hydrogen peroxide (H₂O₂) into water and oxygen.^[^ ^49a,b^ ^]^ Simultaneously, Lorentz forces from AMF‐induced currents create micro‐convective flows, enhancing substrate delivery and product removal, which is critical for diffusion‐limited reactions.^[^ ^49c,d^ ^]^ Additionally, AMFs may alter the spin state of catalase's heme iron, potentially optimizing substrate binding, while nanoparticle surface effects and field‐driven displacement further amplify efficiency.^[^ ^49e,f^ ^]^

### 2.4 In Vitro Study and the Differentiation of Neural Stem Cells (NSCs) by SOCO

To assess the biocompatibility of MC, CAT‐MB, and SOCO, a coculture experiment was performed. Confocal laser scanning microscope (CLSM) images in Figure S5a in the Supporting Information show the interaction between various materials and the NIH‐3T3 cell line, derived from mouse embryonic fibroblasts, after 3 and 7 d of coculture. The results demonstrated that all MB‐based materials supported effective cell attachment on the beads. Over time, an increase in cell numbers was observed. Notably, the presence of CAT led to a higher cell density compared to other groups. Furthermore, in Figure S5b,c in the Supporting Information, cell viability remained above 90% when NIH‐3T3 cells were incubated with MC and SOCO. No significant cytotoxicity was observed for MC at concentrations below 50 µg mL^−1^. However, when the concentrations of MoCx‐Cu increased to 100 µg mL^−1^, the cell viability decreased to 80%. Embedding the particles in MB reduced the toxicity (Figure S5c in the Supporting Information), and SOCO demonstrated good biocompatibility as well.

Neural stem cells (NSCs) are crucial for brain regeneration and plasticity, making them a key focus of this study, which aimed to evaluate the effects of various materials on their function. Herein, we demonstrate to cultivate NSC in vitro and the extraction process is detailed in Figure S6a,b in the Supporting Information, with differentiation beginning by day 7. The experiment included five groups: (1) Normal NSCs as the control, (2) MB, (3) CAT‐MB, (4) SOCO, and (5) SOCO+AMF. In the AMF‐treated group, a magnetic field with a power of 3.2 kW and a frequency of 1 MHz was applied daily for 5 min until the NSCs were immobilized. To investigate the cell viability of nerve‐related cell lines, neural stem cells (NSCs) were used as a model. The cell viability of NSCs remained above 92% when incubated with MC and SOCO, indicating no significant cytotoxicity (Figure S6c,d, Supporting Information). At concentrations of MC below 50 µg mL^−1^, no notable toxic effects were observed. However, when the concentration of MC increased to 100 µg mL^−1^, cell viability dropped to 70%. GFAP and MAP‐2 expressions were used as markers for astrocytes and neurons, respectively. Figure S7a in the Supporting Information shows good cell viability and differentiation across all groups (Figure S7b; Supporting Information). Axon length measurements using ImageJ in Figure S7c in the Supporting Information indicated that SOCO+AMF enhanced NSC axon growth. Additionally, the area of MAP‐2 expression was notably larger in the SOCO+AMF group compared to the other groups (Figure S7d, Supporting Information).

MAP‐2 staining is appropriate for quantifying axonal growth, as it is not exclusively a dendritic marker but also plays a role in early axonal development and regeneration. Although MAP‐2 is predominantly known for dendritic localization, it is expressed in axons during initial outgrowth and in regenerating neurons, where it helps stabilize microtubules essential for elongation.^[^ ^50a,b^ ^]^ In cultured neurons or injury models, MAP‐2 redistributes to axonal compartments, correlating with the dynamics of axonal growth.^[^ ^50c^ ^]^ This dual localization makes MAP‐2 a viable marker in situations where distinctions between axons and dendrites are less clear, such as in early‐stage development or regeneration. While more specific axonal markers like SMI‐312 or NF200 are available,^[^ ^50d^ ^]^ MAP‐2's broader microtubule‐binding profile offers valuable insight into cytoskeletal changes across both axonal and dendritic compartments, enriching the overall analysis.

Oxygen generation enhances neuron differentiation by improving cellular respiration, which increases ATP production and supports energy‐demanding processes like neurite outgrowth and synapse formation. Additionally, oxygen reduces hypoxia‐induced stress, creating a more favorable environment for neuronal differentiation. Electron stimulation further promotes differentiation by enhancing cellular signaling pathways, such as those involving voltage‐gated ion channels and growth factor receptors, which are crucial for the maturation and functional specialization of neurons. Together, our data supported that SOCO+AMF facilitate electric stimulus and oxygen generation enhances these factors contribute to more efficient and robust neuron differentiation.

### 2.5 In Vivo Angiogenesis and Neurogenesis Study of SOCO on Day 7

A short‐term in vivo study of angiogenesis and neurogenesis following traumatic brain injury (TBI) is important because it helps us understand the brain's early repair mechanisms. After a TBI, the brain needs to quickly restore blood flow through angiogenesis to supply oxygen and nutrients to the injured area. To explore the potential of SOCO for improving TBI recovery, female 7‐weeks old C57BL/6 mice were used in the further study. Traumatic brain injury (TBI) model was created by using a flat‐end puncher to remove brain tissue (Figure S8a, Supporting Information). The materials were implanted in situ right after the brain tissue was removed. After the surgery, an AMF was applied to mice until they were sacrificed on postsurgery days 7, 14, and 42. Immunohistochemistry staining of the harvested brains was conducted. Mice sacrificed on post‐surgery day 7 were analyzed for short‐term recovery and divided into five groups: (1) untreated, (2) MB, (3) CAT‐MB, (4) MC‐MB+AMF, (5) SOCO, and (6) SOCO+HFMF. Furthermore, the immune response of treatments targeting microglia were evaluated using ionized calcium‐binding adaptor molecule‐1 (Iba‐1) staining. Both the untreated and material‐treated groups displayed an obvious presence of microglia (Figure S8b, Supporting Information). However, the mice treated with SOCO showed a marked reduction in Iba‐1 expression, indicating that SOCO effectively diminished the immune response. It is attributed to the release of oxygen, lowering reactive oxygen species (ROS) levels in the microenvironment. Additionally, the group treated with SOCO without AMF had ≈1.8% more microglia compared to those treated with AMF, suggesting that the combination of SOCO and AMF further suppresses the immune response. This approach not only decreases apoptosis but also mitigates harmful factors in the microenvironment. Furthermore, the angiogenesis was enhanced by CAT‐MB and SOCO, suggesting that the controlled and sustained oxygen release played a crucial role in promoting vascular formation. The presence of CAT facilitated the decomposition of hydrogen peroxide into oxygen and water, thereby increasing local oxygen availability. This improved oxygenation likely contributed to endothelial cell proliferation, migration, and new blood vessel formation.

To assess the actual oxygen production of SOCO in brain tissue and confirm its in vivo efficacy, SOCO was implanted into the brain. A fiberoptic pO₂ probe was inserted into brains to monitor oxygen release kinetics during SOCO and SOCO+AMF treatment using the OxyLite 2000 system,^[^ ^51^ ^]^ enabling real‐time measurement of local oxygen dynamics. Notably, normalized TIB pO₂ levels significantly increased for up to 30 min following SOCO+AMF treatment (Figure S9, Supporting Information). Additionally, pO₂ levels in the TBI region rose from 16 ± 2 mmHg to 21.5 ± 3 mmHg over a 60‐min period. These results demonstrate that SOCO+AMF effectively enhances local oxygen availability in brain tissue.

Astrocyte morphology was assessed using GFAP (glial fibrillary acidic protein) staining. Compared to the untreated group, those treated with MB, SOCO, and/or HFMF displayed fewer and less densely packed astrocytes, as shown in Figure S8b in the Supporting Information. The reduced GFAP expression observed following these treatments may be linked to the hydrogel's microporosity. This reduction in astrocyte presence within the trauma site contributed to improved tissue recovery. Neurofilament regeneration was assessed by the axonal marker Neurofilament 200 (NF200), with the SOCO+AMF group displaying higher NF200 expression than other groups (Figure S8b, Supporting Information). MC‐MB+AMF enhanced NF200 expression at day 7, 14 and day 42, suggesting that electron stimulation in vivo promotes neuronal growth. The application of a magnetic field stimulated the generation of electric currents by MC, thereby promoting nerve cell growth.

### 2.6 In Vivo Study of SOCO on TBI on Day 14 and Day 42

After 14 d, a notable decrease in immune responses was observed, with the untreated group showing a more pronounced response than other groups, as depicted in Figure S10 in the Supporting Information. The SOCO group exhibited a roughly 1.6% reduction in Ibal areas compared to the untreated group, highlighting the sustained attenuation of immune responses in the wound microenvironment due to the slow release of catalase from MB. Notably, significant findings were also revealed in the GFAP‐labeled morphology of astrocytes. The untreated group had a higher density of astrocytes, leading to scarring, whereas the MB, CAT‐MB, SOCO, and SOCO+AMF groups displayed fewer and less densely packed astrocytes, with a GFAP area ≈3% lower than that of the untreated group. This reduction in astrocyte scarring is crucial, as it allows for the infiltration of new cells, potentially preventing the formation of larger cavities at the lesion sites and minimizing the risk of losing brain function. Thus, SOCO treatment demonstrated beneficial effects in the context of TBI by reducing astrocyte presence at the wound site.

Assessment of wound healing using vessels labeled with the CD31 marker revealed unique vascular cell morphology in the CAT‐loaded group. In addition, the increase in the number of neurons at the wound site in the SOCO+AMF group was particularly obvious. This group showed a higher number of neurons and a quantified neuronal area was about 3% than the other groups. The ability to stimulate nerve cell growth is critical in treating TBI and has important implications for subsequent functional recovery.^[^ ^5^ ^]^ In addition, our data show magnified images of the brain after MB and SOCO+AMF treatment, as shown in Figure S11a,b in the Supporting Information, further illustrating the results after 7 and 14 d. By day 14, the SOCO+AMF group showed clear and well‐defined vascular and neuronal growth. This enhanced vascularization and neuronal proliferation underscores the potential of SOCO+AMF treatment to promote tissue regeneration and repair. Magnetic fields can induce charge changes that enhance cellular activities, including angiogenesis and neurogenesis, accelerating wound healing and functional recovery.

The extended evaluation period of 42 d revealed a continued reduction in immune responses, with the SOCO+AMF group showing lower quantitative values of Iba1 and GFAP markers compared to the untreated group (Figure S12, Supporting Information). This sustained decrease in immune response indicates the prolonged effectiveness of CAT‐MB in reducing inflammation within the wound microenvironment. In terms of wound recovery, detailed analysis of vascular and neurofilament morphology on Day 42 demonstrated superior outcomes in the SOCO+AMF group compared to other experimental groups. The extended exposure to the high‐frequency magnetic field, combined with catalase‐loaded microspheres, led to enhanced therapeutic effects, including reduced immune responses and improved tissue regeneration. These findings highlight the long‐term benefits of this therapeutic approach in treating traumatic brain injury, emphasizing its potential to promote lasting recovery and functional restoration.

### 2.7 Whole‐Brain Imaging of Blood Vessels and Regenerating Nerve Fibers on Day 42

**Figure 5:**
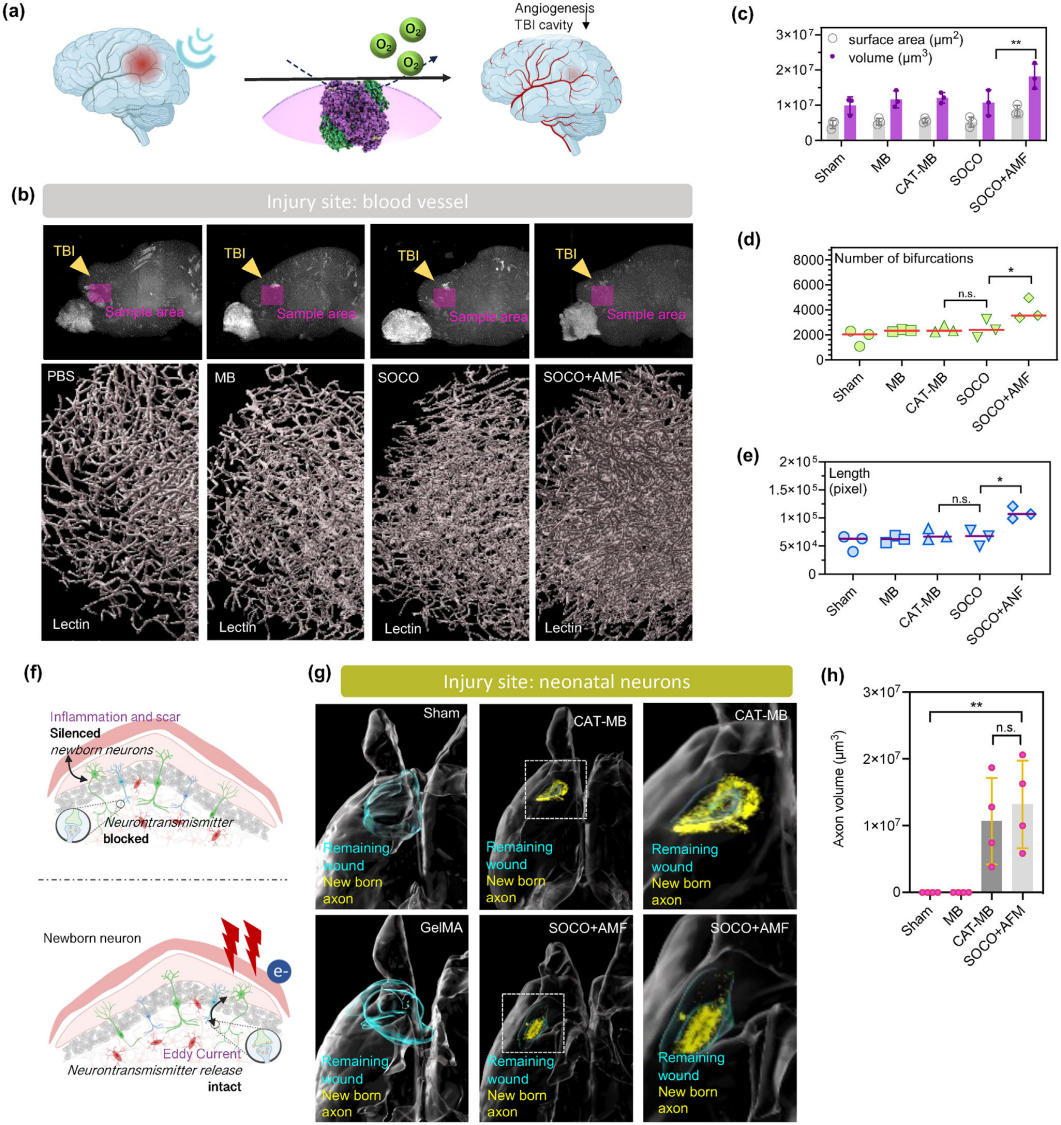
Whole‐brain imaging of blood vessels and neonatal neurons. a) The scheme illustrates oxygen generation and electrical stimulation for blood vessel growth. Whole‐brain images show the area and size of TBI 42 d after various treatments. b) Reconstructed 3D images of blood vessels near the injury site. c–e) Quantification of the surface area, number of bifurcations, and length of blood vessels at the TBI site. (*n* = 5, mean ± s.d., *p < 0.05, **p < 0.01, one‐way ANOVA with Tukey's multiple comparison test.) f,g) Reconstructed 3D images of newborn neurons at TBI site. Electric stimulation (eddy currents) enhances neurotransmitter release to improve neonatal neuronal recovery from TBI. h) Quantification of axon volume around TBI. (*n* = 5, mean ± s.d., **p < 0.01, one‐way ANOVA with Tukey's multiple comparison test.)

To further understand the effect of electron and oxygen generation on angiogenesis (**Figure** **5**a), whole brain‐clearing was conducted and then the blood vessels were labeled with lectin (a protein that has a strong affinity for glycans and therefore is used to label blood vessels). The blood vessels around the injured site of mice treated with SOCO+AMF exhibited a higher density of vessels around the damaged area compared to PBS groups (Figure 5b; Figure S13 and Movie S1, Supporting Information). Statistics also shows that the volume, surface area, number of bifurcations and length of blood vessels have significant increase following SOCO+AMF treatment (Figure 5c,e). These findings suggest that electron and oxygen generation positively influence angiogenesis at the injury site. Electrical signals could potentially activate endothelial ion channels, such as TRP channels, inducing calcium influx to promote proliferation and migration,^[^ ^52a^ ^]^ or upregulate vascular endothelial growth factor (VEGF), as demonstrated by Zhao et al., who reported that electric fields of 75–100 mV mm^−1^ stimulate VEGF production via VEGFR2 signaling, driving tube formation.^[^ ^52b^ ^]^ Oxygen may synergize with this process by stabilizing HIF‐1α to further elevate VEGF levels under hypoxia,^[^ ^52c^ ^]^ a mechanism potentially amplified by electrical stimulation. Other supporting evidence also indicated electrical stimulation to angiogenesis through VEGF‐mediated pathways.^[^ ^52d,e^ ^]^

As shown in Figure 5f, electric current stimuli reduce chronic inflammation that often blocks neuron regeneration and impairs neuronal function, while promoting NSC proliferation and differentiation. To examine neuron regeneration on a whole‐brain scale, we stained the mouse brains with SMI312, a pan‐neurofilament marker. After 42 d post‐TBI, the SOCO+AMF group exhibited significantly increased peri‐wound axon growth and decreased wound volume (Figure 5g,h; Figure S14 and Movie S2, Supporting Information). These findings highlight SOCO+AMF's efficacy in promoting axonal growth, enhancing endothelial proliferation, and facilitating overall neural recovery following injury.

### 2.8 Post‐TBI GABA Signaling

The decrease in GABA inhibitory signals is a key indicator of the critical window for neuronal recovery after a stroke.^[^ ^53^ ^]^ In past traumatic brain injury (TBI) research, post‐TBI GABA upregulation has been observed in patients, suggesting these inhibitory signals negatively impact functional recovery.^[^ ^54^ ^]^ However, inhibitory GABA‐releasing interneurons maintain the balance between excitation and inhibition in cortical circuits. This equilibrium is crucial for brain function, preventing excessive excitation while allowing effective signal transmission.^[^ ^55^ ^]^ For instance, somatostatin (SST) modulates neuron excitability, and its expression significantly decreases in neuropsychiatric disorders.^[^ ^56^, ^57^ ^]^ Research has also shown that SST interneurons are involved in complex motor learning and execution.^[^ ^58^ ^]^ Additionally, studies demonstrate that calbindin in Purkinje cells fine‐tunes rapid calcium signaling, ensuring precise motor coordination and sensory processing necessary for coordinated movements.^[^ ^59^, ^60^ ^]^ These findings illustrate the complex effects of the GABA signaling system on motor function, underscoring the intricate balance required for optimal brain recovery and performance.

**Figure 6:**
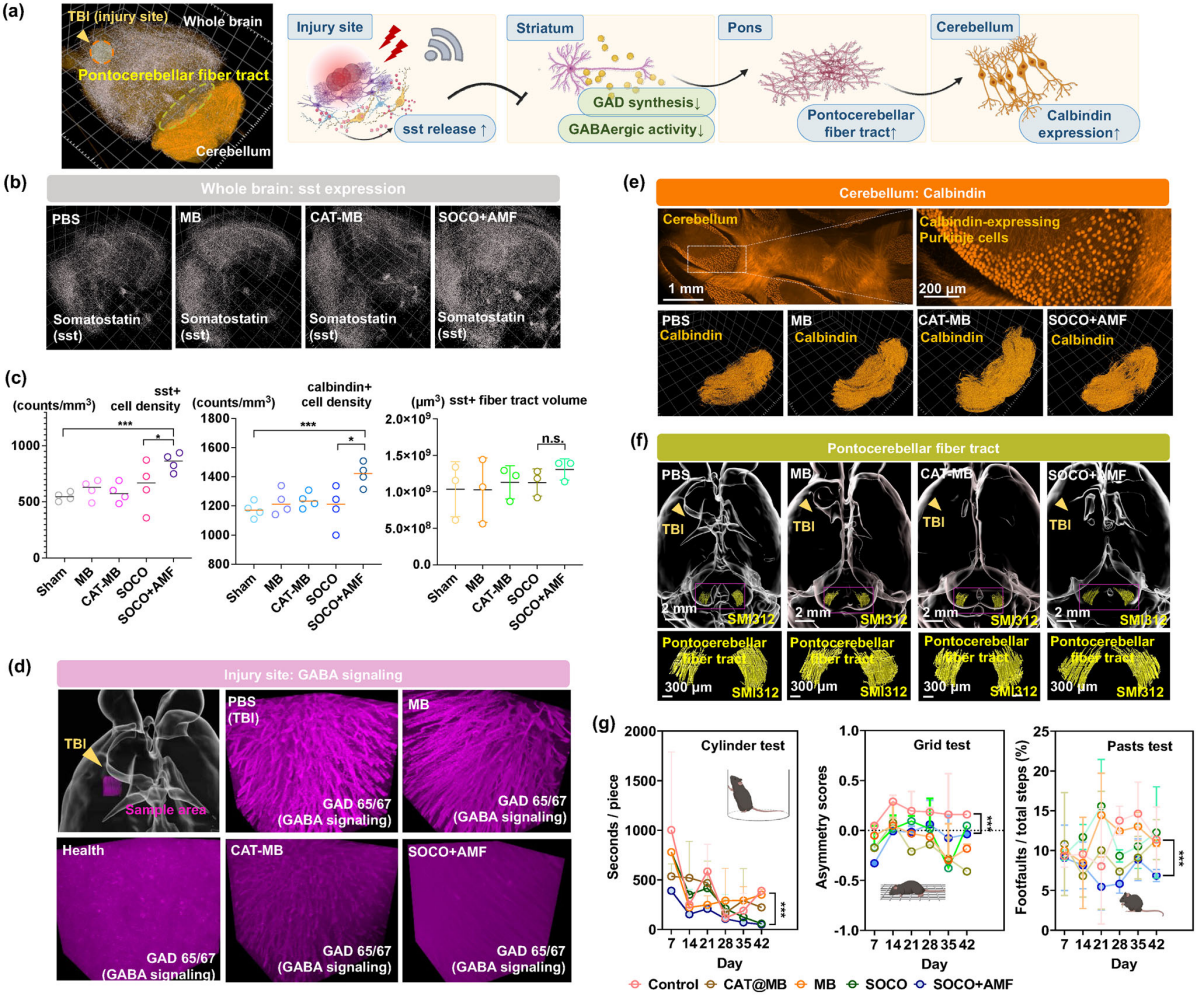
Whole‐brain imaging analyses and behavioral assessments following various treatments for TBI. a) The scheme illustrates the effects of electric stimulation and oxygen at the injury site, brain, and cerebellum. At the injury site, increased SST levels guide the reduction of GAD synthesis and a decrease in GABAergic activity in the striatum. This synergistic effect promotes the growth of photocerebellar fiber tracks and also influences calbindin expression in the cerebellum. b) Reconstructed 3D images of sst and GABAergic expression in the whole brain and at the injury site. c) The quantification of sst, calbindin, and fiber tract volume. d) Reconstructed 3D images of GABAergic expression in the whole brain and at the injury site. (*n* = 5, mean ± s.d., *p < 0.05, ***p < 0.005, one‐way ANOVA with Tukey's multiple comparison test). e) The calbindin expression in cerebellum. f) Reconstructed 3D images of pontocerebellar fiber tract after various treatment. g) Behavioral tests (cylinder, grid, and pasta tests) following various treatment conditions. (*n* = 5, mean ± s.d., ***p < 0.05, one‐way ANOVA with Tukey's multiple comparison test.)

To investigate the recovery of motor function after traumatic brain injury and its potential mechanisms, we conducted whole‐brain staining for several GABAergic neuronal and interneuronal markers including GAD65/67, somatostatin (Sst), and calbindin, and observed the proteomic changes post‐treatment (**Figure** **6**a). We found that in the SOCO+AMF group, cortical and striatal Sst expression significantly increased Figure 6b,c and Figure S15 and Movie S3 the in Supporting Information), while GAD65/67, the marker of GABAergic neurons, in the striatum significantly decreased to levels similar to healthy mice (Figure 6d; Figure S16 and Movie S4, Supporting Information). The number of calbindin‐expressing Purkinje cells in the cerebellum also notably increased after treatment (Figure 6c,e). We also observed that in the SOCO+AMF group, the pontocerebellar fiber tract on the injured side increased compared to other groups (Figure 6f). These protein expression changes collectively indicate improved motor function, aligning with our behavioral assessment results (Figure 6g).

Based on these observations, we propose a hypothetical model (Figure 6a): Increased cortical and striatal SST downregulates inhibitory GABA signaling, leading to enhanced neuron fiber regeneration in the pontocerebellar pathway and increased calbindin‐expressing Purkinje cells. This cascade potentially results in improved motor function. However, it is important to note that this hypothesis requires further experimental validation to establish its validity and explore the underlying mechanisms in greater detail.

### 2.9 Animals’ Behavior Tests

To evaluate recovery and brain function following trauma and treatment, behavioral tests were conducted on female C57BL/6 mice (7 weeks old) divided into five groups: (1) Untreated, (2) MB, (3) CAT‐MB, (4) SOCO, and (5) SOCO+AMF. Each group consisted of four mice, and behavioral experiments were performed weekly over a 42‐d period to assess the long‐term effects of treatment. The cylinder test, which focuses on forelimb movement, was used to assess the impact of brain trauma on limb usage and asymmetry (Figure 6g). Positive and negative values indicate preferences for the right and left limbs, respectively. Results showed that the untreated group's performance deteriorated over time, increasingly deviating from the zero value. To evaluate hindlimb movements, a grid test was conducted to count errors made by the hindlimbs. The untreated group exhibited the highest number of errors, while the SOCO+AMF group made fewer errors compared to the other groups. The pasta test result used for finger dexterity assessment showed that untreated mice took the longest time to consume pasta. Notably, the SOCO+AMF group had the shortest time to finish pasta on post‐surgery day 42, indicating improved finger dexterity.

### 2.10 The Deconvolution of Spatial Data Sets of SOCO Treatment

**Figure 7:**
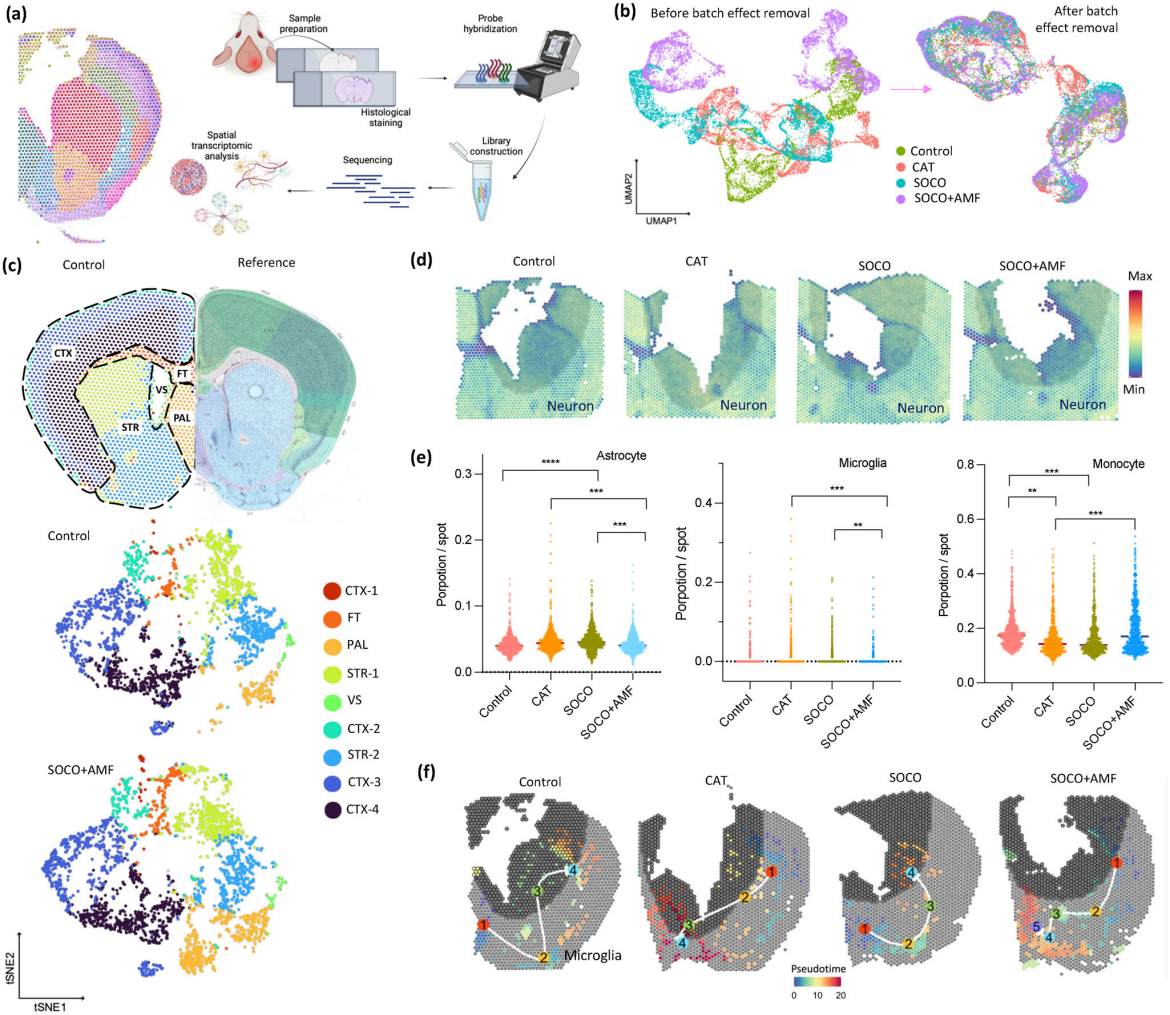
Deconvolution of spatial data sets revealed SOCO treatment in TBI. a) The process of preparing the transcriptional landscape of nanoparticle‐treated TBI mouse brains for spatial data deconvolution analysis. b) Normalization of single‐cell gene of spatial datasets for batch effect removal through SCTransfrom. c) Nine identical brain regions were categorized into control and SOCO+AMF groups. d) Distribution of neuronal cells in the periphery of TBI. e) The quantification of proportion per spot of astrocytes, microglia, and monocytes surrounding the TBI site. (*n* = 5, mean ± s.d., **p < 0.01, ***p < 0.005, ***p < 0.00 one‐way ANOVA with Tukey's multiple comparison test.) f) Trajectory analysis and validation of microglia activation in a mouse model of traumatic brain injury (TBI).

To investigate the transcriptional landscape changes in nanoparticle‐treated traumatic brain injury (TBI) mouse brains, an experiment was designed to compare the control, CAT, SOCO, and SOCO+AMF treatment groups using the Visium CytAssist platform (10x Genomics) (**Figure** **7**a). The goal of this analysis was to map and quantify gene expression changes across different regions of the brain following various treatments, providing spatially resolved insights into how nanoparticles influence the brain's transcriptional response to injury.

In this experiment, brain slides were prepared for each treatment group, with each slide containing ≈14 000 barcoded spots. Each spot had a diameter of 55 µm and included probes designed for capturing the whole transcriptome, enabling high‐resolution mapping of gene expression across the brain. The sequencing results revealed a substantial dataset, capturing an average of 6268 median genes per spot. The breakdown by treatment group was as follows: the control group yielded 6955 median genes per spot, the CAT‐treated group 4973, the SOCO‐treated group 6284, and the SOCO+AMF‐treated group 6863. These differences in gene capture across groups reflect variations in the transcriptional activity elicited by the different treatments.

To ensure data comparability across samples, spatial transcriptomic datasets were normalized for batch effect removal using the SCTransform algorithm (Figure 7b). After normalization, the control dataset was further validated using a k‐means clustering algorithm, which identified 9 distinct clusters based on transcriptional profiles. These clusters were then annotated according to the Allen Mouse Brain Atlas reference, a comprehensive resource for mouse brain anatomy and gene expression (https://mouse.brain‐map.org/static/atlas). The clusters corresponded to specific brain regions, including several cortical areas (CTX1, CTX2/3, CTX4, CTX5, and CTX6), the nucleus accumbens (ACB), lateral ventricles (VL), fiber tracts, and six additional anatomical regions.

The 9 distinct brain regions identified in the control group were consistently categorized across the CAT, SOCO, and SOCO+AMF treatment groups, demonstrating the reliability of gene expression patterns across all four experimental conditions (Figure 7c; Figure S17 in the Supporting Information). This consistency indicates that the spatial transcriptomic data accurately reflects the gene expression dynamics within specific brain regions, allowing for meaningful comparisons between treatment groups. After conducting an initial quality check of the spatial sequencing data, the focus shifted to investigating the changes in cell type composition in response to nanoparticle treatment, specifically in the areas surrounding the injury site. To achieve this, the gene expression captured in Visium spots adjacent to the injured brain tissue was deconvoluted using single‐cell RNA sequencing (scRNA‐seq) data from the Mouse Cell Atlas (MCA) (http://bis.zju.edu.cn/MCA/). Notably, the SOCO+AMF treatment led to distinct changes in cellular composition near the injured area (Figure 7d; Figure S18 in the Supporting Information). Compared to the CAT and SOCO‐treated groups, the SOCO+AMF‐treated brains exhibited a significant decrease in astrocytes and microglia—two cell types often associated with neuroinflammation and injury response (Figure 7e). Collectively, our findings support that the SOCO+AMF treatment not only reduces neuroinflammation but also alters the neuronal composition, potentially contributing to its therapeutic effects in the context of TBI.

Notably, the proportion of monocytes is significantly increased in the SOCO+AMF‐treated samples, highlighting a possible therapeutic response linked to enhanced immune surveillance or repair mechanisms at the site of injury (Figure 7e; Figure S19 in the Supporting Information). In contrast, the proportions of granulocytes and ependymal cells remain consistent across all groups, suggesting that these cell types are not differentially affected by the treatments. Interestingly, the proportion of oligodendrocytes displays a dual pattern: SOCO+AMF treatment results in a decrease in oligodendrocyte numbers when compared to CAT treatment but an increase relative to the control and SOCO groups. This may indicate a nuanced effect of the combined SOCO+AMF therapy on myelination or oligodendrocyte dynamics, which are critical for maintaining and repairing neural function. The contrasting changes in oligodendrocyte populations hint at a potential role of the treatment in modulating glial cell activity, which could be important for long‐term brain repair processes. The observed changes in cell populations around the injured area provide valuable insights into how nanoparticle treatments can modulate the brain's cellular response to injury.

Additionally, we traced the cell lineage based on the directionality of transcriptional alternation of microglia through the pseudotemporal reconstruction tool. The spatial trajectory of microglia activation of control, CAT, and SOCO treatment groups was from the uninjured area (node 1) to the injured region (node 4), and particularly, the microglia surrounding the damaged region were not activated in SOCO+AMF‐treated brain (Figure 7f). Altogether, these findings pointed out reduced cell population and lack of recruiting of microglia to the injury site in the SOCO+AMF‐treated brain. To deepen the understanding of these cellular shifts, single‐cell RNA sequencing (scRNA‐seq) data was integrated with spatial datasets, overcoming the inherent resolution limitations of spatial transcriptomics. The deconvolution of these datasets provided crucial insights into the immune landscape of the treated brain tissues. Specifically, the SOCO+AMF treatment appears to mitigate the inflammatory response, as evidenced by a reduced proportion of microglia and astrocytes, two cell types commonly associated with neuroinflammation and scarring after brain injury.^[^ ^61^, ^62^ ^]^ Microglia and astrocytes, when activated, often exacerbate neuroinflammation, leading to secondary damage. Therefore, the observed reduction in these cell populations suggests a dampening of the inflammatory milieu, potentially fostering a more favorable environment for healing.

Moreover, the elevated presence of monocytes in the SOCO+AMF‐treated brains likely play a pivotal role in wound healing by aiding in the clearance of cellular debris and dead cells at the site of damage. Monocytes are key players in the early stages of repair, as they contribute to debris phagocytosis and secretion of factors that promote tissue regeneration. The combination of reduced neuroinflammatory cells and increased monocyte‐mediated clearance might synergistically enhance the brain's natural repair processes. Thus, the SOCO+AMF treatment seems to induce a shift towards a reparative immune response, supporting tissue recovery while minimizing the adverse effects of chronic inflammation.

### 2.11 The SOCO Treatment Promotes Angiogenesis and Neurogenesis

**Figure 8:**
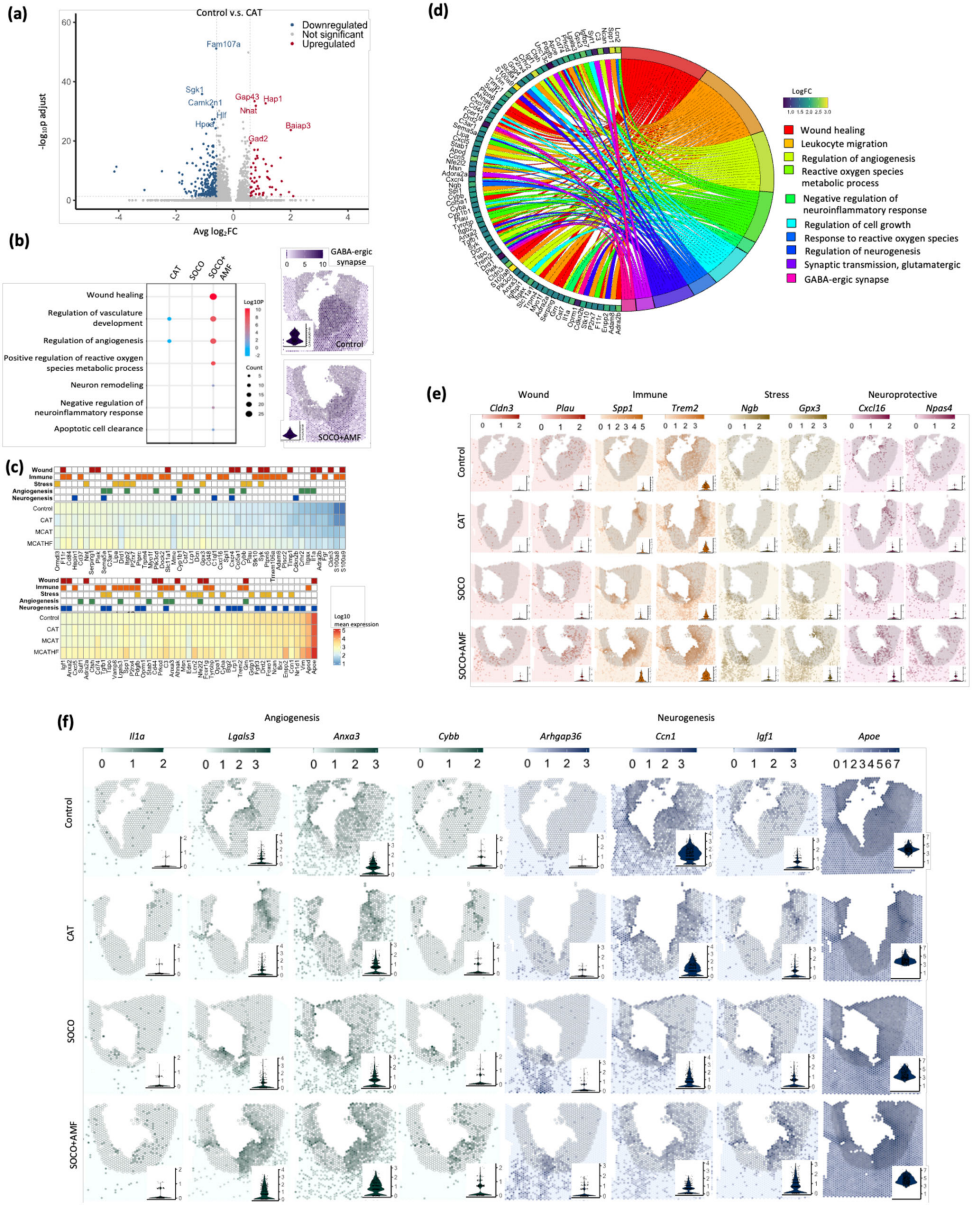
Functional gene analysis of spatiotemporal transcriptomics in response to various treatments. a) Volcano plot showing differentially expressed genes in CAT group compared to control. Red dots represent upregulated genes, blue dots represent downregulated genes, and grey dots represent nonsignificant genes. b) Left, dot plot of enriched biological processes based on differentially expressed genes, highlighting processes such as wound healing, regulation of angiogenesis, and apoptotic cell clearance. Color indicates significance (Log10P) and size represents gene count. Right, the spatial pattern of sum of gene expression related to GABAergic synapse neighboring injury site. c) Heatmap of gene expression profiles across samples, displaying hierarchical clustering of samples and genes based on expression levels. d) The chord graph reveals the relationship between genes and specific GO terms. e,f) Spatial gene expression maps for selected genes across various brain regions, visualized across sequential sections of the brain. Heatmap scales at the top indicate gene expression levels.

The deconvolution of spatial datasets suggested that different treatments triggered varying degrees of cellular distribution following brain injury. Given this, we proceeded to examine the spatial transcriptomic data itself for deeper insights. To uncover the gene expression changes associated with each treatment, we identified differentially expressed genes (DEGs) by comparing treatment groups to the control (**Figure** **8**a). This analysis provided a foundation for understanding how each treatment modulates the brain's transcriptional landscape in response to injury. To further elucidate the impact of the treatments on cellular mechanisms, we performed gene ontology (GO) analysis on the identified DEGs. This analysis revealed key biological processes that were upregulated in the SOCO+AMF‐treated injured brains. These included pathways related to wound healing, regulation of angiogenesis, neuronal remodeling, and the negative regulation of neuroinflammatory responses (Figure 8b). These processes are crucial for tissue repair and recovery following traumatic brain injury, suggesting that the SOCO+AMF treatment may promote a more robust regenerative response. In contrast, the regulation of angiogenesis was found to be downregulated in CAT‐treated samples, indicating a potential impairment in the vascular response to injury in this treatment group. Interestingly, no GO categories related to these repair processes were significantly up‐ or downregulated in the SOCO‐treated brains, highlighting a distinctive effect of the SOCO+AMF combination therapy that was absent in SOCO treatment alone. Furthermore, both GABAergic synapse activity and cerebral cortex GABAergic interneuron differentiation showed downregulation, consistent with observations from whole brain imaging, and reduced glutamatergic synapse (Figures 6 and 8b; Figure S20a in the Supporting Information). The reduced GABAergic synapse activity in the SOCO+AMF treatment group suggests that this therapeutic approach modulates GABA synthesis, potentially enhancing the brain's intrinsic repair mechanisms. This finding supports the proposed role of GABA in maintaining a balanced excitatory–inhibitory environment, which is crucial for effectively facilitating nerve regeneration.

To visualize the spatial distribution of genes associated with the observed GO terms, we mapped the expression of these genes onto the brain tissues of the different treatment groups. The spatiotemporal transcriptome expression of wound healing and neurotransmitter transport genes in the injured region of the brain treated with SOCO+AMF was significantly increased and decreased, respectively, compared with other groups (Figure 8b; Figure S20b, Supporting Information). This spatial enrichment further supports the idea that the SOCO+AMF treatment uniquely elevates the expression of genes involved in wound healing, angiogenesis, and neuronal remodeling, while also modulating neuroinflammatory pathways and excitatory–inhibitory environment. These findings indicate that the SOCO+AMF treatment, unlike CAT and SOCO treatments, enhances the expression of genes linked to critical processes such as tissue repair, vascular formation, and neuronal restructuring. Additionally, it appears to downregulate genes involved in neuroinflammation and neurotransmitter releasing, which is often associated with secondary damage following brain injury. Altogether, this suggests that SOCO+AMF may create a more favorable environment for brain healing by both promoting regeneration and mitigating harmful inflammatory responses.

The gene expression profiles of genes involved in wound healing, immune response, stress, angiogenesis, neurogenesis, and GABAergic and glutamatergic synapse, based on GO categories, were visualized through a heatmap to compare the treatment effects across different samples and a chord diagram to show the association of GO terms and related DEGs found in SOCO+AMF treatment group (Figure 8c,d). Notably, certain genes displayed distinct trends of either increasing or decreasing expression between treatment groups, such as ***Cldn3***, ***Apoe***, ***S100a8***, ***Ccn1***, and ***Il1a***. These trends were further analyzed by mapping their spatial expression around the injured brain regions across all experimental groups (Figure 8e,f). In the wound healing category, ***Cldn3*** and ***Plau*** were upregulated in the SOCO+AMF (MCATHF)‐treated injured brains, indicating enhanced wound healing and tissue closure processes. This is supported by previous findings linking these genes to the promotion of barrier integrity and fibrinolysis, key mechanisms in tissue repair.^[^ ^63^, ^64^ ^]^ Additionally, the SOCO+AMF treatment group showed increased expression of ***Spp1*** and ***Trem2***, both of which are associated with the release of anti‐inflammatory cytokines, suppression of neural apoptosis, and mitigation of inflammatory responses, further emphasizing the anti‐inflammatory effects of this treatment.^[^ ^65^, ^66^ ^]^ Furthermore, genes related to cellular stress and neuroprotection, such as ***Ngb*** and ***Gpx3***, as well as ***Cxcl16*** and ***Npas4***, were also upregulated in the SOCO+AMF‐treated group. This suggests that the treatment reduces oxidative stress and enhances neuroprotective mechanisms, contributing to improved outcomes after brain injury.^[^ ^67^, ^68^ ^]^

Additionally, markers of angiogenesis, such as ***Il1a***, ***Lgals3***, ***Anxa3***, and ***Cybb***, were elevated in the SOCO+AMF‐treated samples. These genes are involved in endothelial cell activation, migration, proliferation, and tube formation, processes essential for repairing damaged blood vessels and promoting tissue regeneration.^[^ ^69^, ^70^ ^]^ In parallel, the upregulation of neurogenesis‐related genes, including ***Arhgap36***, ***Igf1***, and ***Apoe***, along with the downregulation of ***Ccn1***, indicated the activation of neural stem cell proliferation and differentiation, suggesting enhanced neuronal development and recovery following injury in the SOCO+AMF group.^[^ ^71^, ^72^ ^]^ The transcriptional changes induced by SOCO+AMF treatment were multifaceted, promoting key biological processes such as wound healing, angiogenesis, neurogenesis, and the metabolism of reactive oxygen species while dampening inflammatory responses. These molecular changes likely contribute to the improved recovery and tissue repair observed with this treatment, differentiating it from the effects of CAT and SOCO treatments alone.

The gene ontology (GO) analysis and spatial visualization of gene expression patterns provided critical insights into the cellular mechanisms and biological processes occurring in the damaged brain regions in response to different treatments. Most notably, the results highlighted the activation of neural development pathways in SOCO+AMF‐treated brain tissues compared to control, CAT, and SOCO‐treated samples. Despite a reduction in the overall proportion of neurons in the SOCO+AMF group, genes associated with neural development were highly expressed, suggesting enhanced neuronal repair and regeneration. It is important to note, however, that these neural development genes were not captured in the adult mouse brain single‐cell data used for deconvolution, which may explain the discrepancy between cellular proportion data and transcriptional activity. Furthermore, the spatial transcriptomics datasets with single‐cell deconvolution analysis supported the aforementioned findings, and provided detailed information on gene expression alternation upon various treatment conditions spatially, including spatial patterning of microglia activation and initiation of angiogenesis and neurogenesis and synaptic transmission genes.

Additionally, the analysis of cellular distribution from deconvoluted spatial data, combined with gene expression analysis, revealed a significant reduction in microglia and astrocytes surrounding the injured area in the SOCO+AMF‐treated brains. This reduction in glial cells is consistent with a suppressed neuroinflammatory response, a finding supported by both cellular and gene‐level data. The convergence of these two lines of evidence strengthens the conclusion that SOCO+AMF treatment effectively mitigates neuroinflammation. Moreover, the gene expression analysis identified key processes promoted by SOCO+AMF treatment, including angiogenesis and wound closure, as well as a reduction in cellular stress. The activation of these pathways likely plays a critical role in tissue repair and recovery, enhancing the overall therapeutic effect of the treatment. Taken together, our findings support that SOCO+AMF treatment fosters a regenerative environment in the injured brain by promoting neuronal development, reducing inflammation, and supporting vascular and tissue repair.

## 3 Conclusions

Current therapeutic approaches for nerve regeneration in traumatic brain injury (TBI) face significant limitations, highlighting the need for alternative strategies like electrical stimulation. Small molecule drugs often suffer from poor target specificity and rapid systemic clearance, reducing their effectiveness in sustained nerve repair. Growth factor delivery, such as NGF and BDNF, is hindered by short half‐lives and diffusion limitations, preventing long‐term therapeutic concentrations. Gene therapies also face challenges, including inefficient transfection, immune responses, and off‐target effects, limiting clinical success. In contrast, electrical stimulation offers a promising solution by providing precise, localized control over nerve regeneration.

In summary, this study presents a novel therapeutic strategy utilizing wireless‐charging sustained oxygen release from conductive microgels (SOCO) for promoting nerve regeneration and brain recovery in TBI. By introducing an “electromagnetic messenger” that combines external alternating magnetic fields (AMF) with catalytic oxygen release and electrical stimulation, the approach reprograms brain transcriptomes, influencing key processes such as angiogenesis and neuronal regeneration. The ability of SOCO to promote somatostatin production and modulate GABA synthesis demonstrates its potential to enhance brain function and behavioral recovery. Furthermore, spatial multiomics combined with single‐cell deconvolution analysis demonstrates that treatment with the SOCO system reprograms the in vivo brain transcriptome, particularly promoting angiogenic (Il1a, Lgals3) and the GABAergic pathway by modulating GAD65/67 activity. This, in turn, promotes angiogenesis and neuronal regeneration. The in situ catalytic SOCO, activated by non‐contact alternating magnetic fields (AMF), introduces an innovative “electromagnetic messenger” therapeutic approach that effectively reprograms neuro‐regeneration and enhances brain function recovery in traumatic brain injury (TBI).

## 4 Experimental Section

### Materials

Gelatin from bovine skin (Type B, Sigma‐ Aldrich, #SI‐G2625), 2‐hydroxy‐4'‐(2‐hydroxyethoxy)‐2‐methylpropiophenone (Irgacure D‐2959, Sigma‐ Aldrich, #AL‐410896‐10G), Paraffin oil, Span 80, Rhodamine B isothiocyanate mixed isomers (RITC, Aldrich‐Sigma), Copper(II) acetate monohydrate, l‐glutamic acid free acid, 1,3,5‐benzenetricarboxylic acid, Ethanol (95%, Aldrich‐Sigma), DMSO, Cy5.5Amdite (GE Healthcare), catalase from bovine liver, Triton X‐100 (J.T.Baker), Penicillin–Streptomycin Solution, Ethanol (95%, Aldrich‐Sigma), Phosphate buffer saline (PBS, ultra‐pure grade, AmRESCO), Dulbecco's Modified Eagle Medium (DMEM), Kaighn's Modification of Ham's F‐12 Medium (F‐12K, Gibc), HBSS, Fetal Bovine Serum (FBS, standard, Gibco), Penicillin–Streptomycin Solution, Trypsin, 2.21 × 10^−3^ m EDTA Solution (0.25%,), Trypan Blue Solution (0.4%, Aldrich‐Sigma), 4′‐6‐Diamidino‐2‐phenylindole (DAPI, Invitrogen), F‐actin (Alexa Fluor 488 phalloidin, Invitrogen), Paraformaldehyde (#158127‐100G), for mammalian cells (ThermoFisher, # L3224), l‐Glutamic acid monosodium salt hydrate ≧99% (HPLC, Powder, Sigma, #G1626‐100G), PrestoBlue cell viability reagent (Thermo, #A13261), Anti‐glial fibrillary acidic protein antibody (anti‐GFAP antibody) (Merck, #AB5804), Mouse Anti‐MAP2 (anti‐microtubule‐associated protein 2) (Sigma, #MAB3418), Mitochondrial Hydroxyl Radical Detection Assay Kit (Abcam, #ab219931), Donkey Anti‐Rabbit Alexa Fluor 647 (Abcam, #ab150075), Goat Anti‐Mouse Alexa Fluor 488 (Abcam, #ab150113), IBA‐1 Polyclonal Antibody (Invitrogen, #PA5‐18039), Anti‐clusterofdifferentiation31(anti‐CD31antibody) (Abcam, #ab124432), Anti‐Neurofilament heavy polypeptide antibody (Abcam, #ab8135), Donkey Anti‐Rat Alexa Fluor 488 (Life, # a21208)

### Fabrication of Microfluidic Chips

The microfluidic chip was designed using geometric modeling software (AutoCAD, Autodesk Inc., Sausalito, CA, USA). The design patterns were then laser‐etched onto a PMMA sheet using a CO_2_ laser micromachining system (LES‐10, Laser Life Co. Ltd., Taiwan) set at 50% power and 15% speed. The chip's final dimensions were 65 mm in length, 35 mm in width, and 4 mm in height. The etched PMMA plates were immersed in deionized (D.I.) water and subjected to ultrasonic agitation for 30 min. Following multiple washes with D.I. water and ethanol, the PMMA sheets and glass panes were heated at 105 °C overnight. Finally, the plates were tightly pressed together, and Polyether ether ketone (PEEK) tubes were affixed into the predrilled holes using AB glue.

### Synthesis of GelMA

First, GelMA was prepared by dissolving 5 g (10% w/v) of type B bovine skin gelatin in 50 mL of deionized water at 50 °C. The mixture was stirred using a magnetic stirrer. Next, 2.5 mL of methacrylic anhydride (MA) was slowly added to the gelatin solution, and the mixture was allowed to react for 3 h. The resulting solution was then dialyzed at 40 °C using tubes with a 12–14 kDa dialysis membrane. The dialysis procedure involved changing the solution with deionized water twice daily for 5 d to remove any unreacted methacrylic anhydride (MA) and salts. Subsequently, the solution was lyophilized for 4 d to remove all water. The resulting lyophilized GelMA had a foamy appearance and was stored at −20 °C for future use.

### Fabrication of MB

The fabrication of microspheres using a microfluidic chip begins with the preparation of the GelMA solution, which serves as the aqueous phase. This solution consists of 3 mL of deionized water, 7.5 wt% GelMA, and 0.5 wt% Iroacure 2529. The CAT‐GelMA solution is similarly prepared, containing 7.5 wt% GelMA in 3 mL of deionized water, with an oil phase comprising 5 wt% Span80 in paraffinic oil. The flow rates of these two phases are carefully controlled using a syringe pump, and the entire setup is placed under an inverted light microscope to monitor droplet formation.

At the microfluidic outlet, the microsphere droplets are crosslinked using UV light (365 nm, 1500 series, OmniCure). The size of the microspheres is determined using microscope images analyzed with Nikon software. Afterward, the microspheres are washed three times with hexane, followed by centrifugation in deionized water to remove any residual oil and surfactant. The upper organic phase is then removed, and the microspheres are stored at −20 °C for future use. It is crucial to perform these experiments at room temperature, above 20 °C, due to the gelling temperature of GelMA.

For fluorescence imaging of microsphere morphology, rhodamine B isothiocyanate (RITC) is used to stain the microspheres red. After fabrication on the microfluidic water‐in‐oil (w/o) chip, the microspheres are collected and washed multiple times with excess deionized water. They are then dispersed in water with 0.01% RITC and stored at 4 °C for 3 d to allow for staining. After staining, the microspheres are washed three times with water to remove any unreacted RITC. Finally, the microspheres are centrifuged to eliminate any remaining dye and stored at −20 °C for future use.

### Synthesis of Metal–Organic Frameworks (NENU5) and MC

The synthesis procedure followed the typical NENU‐5 synthesis method. First, 0.14 g of 1,3,5‐benzenetricarboxylic acid (BTC) was added to 40 mL of 95% ethanol and sonicated for 20 min (Solution A). Meanwhile, prepare 40 mL of deionized water in a 100 mL vial and stir it with a magnetic stirrer at room temperature. Next, 0.2 g of copper(II) acetate monohydrate and 0.073 g of l‐glutamic acid free acid were added to the stirred deionized water and stirred at room temperature for 20 min until completely dissolved (Solution B). The stirring speed should not be too low to avoid precipitation. After 20 min, pour Solution A into Solution B and stir for 14 h. The resulting mixture was washed three times with 95% alcohol using a sonicator and then centrifuged at 6000 rpm for 6 min. The mixture was then transferred to an evaporating dish and placed in a 60 °C oven to dry overnight. Finally, the blue‐green powder obtained in the evaporating dish represents the NENU‐5 particles.

Place the obtained NENU‐5 (0.1 g) into a tube furnace. Purge the tube furnace with argon (Ar) for 30 min to remove any entrapped air. Then, set the temperature to increase by 2 °C every hour. Once it reaches 450 °C, keep it at that temperature for 2 h. Maintain a constant flow of Ar gas at a rate of 80 s.c.c.m. throughout the process. Once the furnace has cooled to room temperature, wash the resulting product three times with 95% ethanol using a sonicator and centrifuge the product at 6000 rpm for 6 min. Transfer the mixture to an evaporating dish and place it in a 60 °C oven to dry overnight. The collected residue will be a black powder, indicating the formation of MC particles.

### Coating MOF and MC on MBs

To prepare 10 mg of microspheres (GelMA or CAT‐MB, depending on the experiment) for filling a post‐traumatic brain injury (TBI) mouse, first measure out 10 mg of microspheres (GelMA or CAT‐MB) and set it aside. Separately, prepare a MOF or MC solution of the desired concentration, for example, 10 µg mL^−1^ of MC. Add the 10 mg of microspheres to the MOF solution and start shaking the mixture continuously for 30 min. During this process, the microspheres change color significantly, indicating that the MOF has attached. Finally, wash the microspheres three times with PBS (Phosphate Buffered Saline). This step helps to remove any excess MOF solution and ensures that the microspheres are clean and ready for the experiment.

### Degradation of MBs

Weigh the microspheres to ensure they are at 10 mg. Add a fixed volume of PBS (phosphate buffered saline). After adding PBS, transfer the microspheres to an incubator set at 37 °C. Incubate the microspheres in PBS for the indicated durations. Remove them from the incubator on days 1, 3, 7, 12, and 14. Carefully remove the PBS solution from the microspheres without disturbing or losing any microspheres. After removing the PBS, proceed to freeze‐dry the microspheres to obtain a powder form. Weigh the lyophilized powder to determine the final weight of the microspheres at each time point (days 1, 3, 7, 12, and 14).

### Embryonic NSCs Extraction Experiments

Euthanize 16‐d pregnant Wistar rats (ED16) using CO_2_, and immediately transfer them to a laminar flow bench for subsequent experiments. Carefully remove the embryos from the mother and wash them with HBSS buffer. Neural stem cells (NSCs) were isolated from the right brain region of the embryos. The extracted brain tissue was minced with forceps and temporarily stored in HBSS buffer.

The cell suspension was then centrifuged at 1000 rpm for two 7‐min cycles. After centrifugation, the cell pellet was resuspended in 5 mL of reconstituted medium, composed of F12/DMEM supplemented with 1% penicillin and 1% N‐2 supplement. To promote cell growth, an additional 5 mL of reconstitution medium and 10 µL of basic fibroblast growth factor (BFGF) were added to the flask. The cells were cultured for 4 d, during which NSCs formed suspended spheroids. These NSC spheroids were maintained in a 37 °C incubator with 5% CO_2_, with the culture medium refreshed weekly to support ongoing growth.

### Differentiation of NSCs Experiment

To ensure adherence of NSCs, slides were coated with poly‐l‐lysine (PLL). The procedure began with cleaning the slides with alcohol to remove surface dirt and grease, followed by exposure to UV light for 15 min. Next, 500 µL of PLL was applied to the surface of the slide and left for 30 min.

Before seeding NSCs on slides, it was recommended to wash them three times with PBS. The number of NSC speroids was determined using a 24‐well plate, with 200 NSC spheroids seeded in each well. Once the NSC spheres firmly attached to the glass slide (≈8 h), various materials could be added to the Petri dish for co‐culture with the NSCs. It is important to note that the differentiation medium promoting NSC differentiation differed from the maintenance medium.

After 7 d, NSCs were washed three times with PBS and fixed with a 70% methanol solution for 5 min. The fixed NSCs were then washed three times. Subsequently, samples were soaked in blocking buffer containing 5% BSA for 1 h at room temperature. After removing the blocking solution, samples were incubated overnight with primary antibodies: (1) rabbit anti‐glial fibrillary acidic protein (GFAP) diluted 1:500, and (2) mouse anti‐microtubule‐associated protein 2 (MAP‐2) at a 1:500 dilution. NSCs were washed three times with PBS every other day and stained with secondary antibodies for 2 h at room temperature. The secondary antibodies used were: (1) donkey anti‐rabbit IgG (H&L) at a 1:500 dilution, and (2) goat anti‐mouse IgG (H+L) antibody (FITC) at a 1:500 dilution. Subsequently, the samples were washed three times with PBS and mounted with DAPI to stain the samples. Finally, observations were performed using a laser scanning confocal microscope (ZEISS LSM‐780). To calculate the percentage of phenotypically differentiated cells, the intensity of GFAP‐ and MAP‐2‐positive cells in the extra neural region was quantified to determine the percentage of astrocytes and neurons in each field of view. Image J software was used for this analysis.

### In Vivo Study of TBI and Postoperative Care

The surgical procedure was conducted in accordance with the protocol approved by the Animal Care and Use Committee of National Tsing Hua University (Approval No. 111063), Hsinchu, Taiwan. Prior to implantation, the microbubbles (MBs) were fully swollen by immersion in phosphate‐buffered saline (PBS) at room temperature. Female C57BL/6 mice (7 weeks old) were randomly divided into four groups (*n* = 6 per group): (1) PBS control, (2) MBs, (3) SOCO, and (4) SOCO + AMF. To induce traumatic brain injury (TBI), mice were anesthetized with an intraperitoneal injection. The skull was exposed via a midline incision under aseptic conditions, and a high‐speed electric drill was used to create a circular cranial window. A 2‐mm‐diameter biopsy punch was then employed to generate a cortical injury of 1.5 mm in depth by removing brain tissue. Following injury induction, 10 µL of MBs or SOCO (with a gel volume fraction of 58% v/v in PBS) was injected using a microsyringe. In the AMF treatment group, alternating magnetic field (AMF) stimulation was applied for 5 minutes per day until the mice were sacrificed.

Postoperative care was provided to ensure animal welfare and minimize discomfort. Mice were placed in a temperature‐controlled recovery chamber until they regained consciousness, after which they were returned to their home cages with access to soft bedding. Buprenorphine (0.1 mg/kg, subcutaneously) was administered every 12 h for 48 h to manage postoperative pain. Mice were monitored daily for signs of distress, neurological deficits, or infection, and their body weight was recorded to assess overall health. To prevent dehydration, subcutaneous saline (0.5 mL) was administered if necessary. The surgical site was inspected regularly, and any signs of infection were treated with appropriate antibiotics. AMF treatment was performed as per the experimental protocol, and animals were observed for adverse reactions. All mice were housed under a 12‐h light/dark cycle with ad libitum access to food and water throughout the study.

### Statistical Analysis

Statistical analyses were conducted using GraphPad Prism software (version 9.0) based on data obtained from three or more independent experiments. Error bars in the figures represent the standard deviation (SD) derived from three or more independent experiments. A one‐way analysis of variance (ANOVA) was initially performed to evaluate differences between groups, followed by either Dunnett's or Tukey's multiple‐comparison tests, as specified in the figure legends. *P < 0.05, **P < 0.01, and ***P < 0.005 were considered statistically significant.

## Conflict of Interest

The authors declare no conflict of interest.

## Supporting information



Supporting Information

Supplemental Movie 1

Supplemental Movie 2

Supplemental Movie 3

Supplemental Movie 4

## Data Availability

The data that support the findings of this study are available from the corresponding author upon reasonable request.;
