# Supplementary material for: Wireless In Situ Catalytic Electron Signaling‐Mediated Transcriptomic Reprogramming for Neuron Regeneration via Adaptable Antennas
Source: Adv Sci (Weinh). 2025 May 11;12(28):2504786. doi: 10.1002/advs.202504786 (PMC12302649; doi:10.1002/advs.202504786)
Supplement: Supplementary file 1 — Supporting Information [file ADVS-12-2504786-s002.pdf]

ADVANCED SCIENCE
Open Access

Supporting Information

for *Adv. Sci.*, DOI 10.1002/advs.202504786

Wireless In Situ Catalytic Electron Signaling-Mediated Transcriptomic Reprogramming for Neuron Regeneration via Adaptable Antennas

Hoi Man Iao, Chih-Ying Chen, Ya-Hui Lin, Wan-Chi Pan, Chun-Yi Liang, Hsiu-Ching Liu, Lo-Jei Ching, Pei-Yu Weng, Min-Ren Chiang, Ru-Siou Hsu, Tsu-Chin Chou, I-Chi Lee, Lun-De Liao, Li-An Chu, Shih-Hwa Chiou* and Shang-Hsiu Hu**

Supporting Information

Wireless *In Situ* Catalytic Electron Signaling-Mediated Transcriptomic Reprogramming for Neuron Regeneration *via* Adaptable Antennas

Hoi Man Iao[1], Chih-Ying Chen[2], Ya-Hui Lin[1,3], Wan-Chi Pan[1], Chun-Yi Liang[1], Hsiu-Ching Liu[1], Lo-Jei Ching[2], Pei-Yu Weng[3], Min-Ren Chiang[1], Ru-Siou Hsu[4], Tsu-Chin Chou[5], I-Chi Lee[1], Lun-De Liao[6], Li-An Chu[1,3,*], Shih-Hwa Chiou[2,7,*], Shang-Hsiu Hu[1,2,5,*]

[1] Department of Biomedical Engineering and Environmental Sciences, National Tsing Hua University, Hsinchu 300044, Taiwan
[2] Department of Medical Research, Taipei Veterans General Hospital and National Yang Ming Chiao Tung University, Taipei 112201, Taiwan
[3] Brain Research Center, National Tsing Hua University, Hsinchu, 300044 Taiwan
[4] Department of Biomedical Engineering, National Yang Ming Chiao Tung University, Taipei, 112304, Taiwan
[5] Institute of Analytical and Environmental Sciences, National Tsing Hua University, Hsinchu, 300044 Taiwan
[6] Institute of Biomedical Engineering and Nanomedicine, National Health Research Institutes, Miaoli County, 35053 Taiwan
[7] Institute of Pharmacology, College of Medicine, National Yang Ming Chiao Tung University, Taipei, 112304 Taiwan.

E-mail addresses: lachu@mx.nthu.edu.tw, shchiou@vghtpe.gov.tw, shhu@mx.nthu.edu.tw

Methods

3D imaging and analysis of whole mouse brain

The mice were euthanized by injecting them with 0.2 mL of a 30% Urethane saline solution. The euthanized mice were perfused with 20 mL of cold phosphate-buffered saline (PBS) and 20 mL of 4% PFA. Whole brains were collected and further fixed using 4% PFA at 4 °C. The PFA-fixed brains were incubated in SHIELD-OFF solution at 4 °C for 96 hours, followed by incubation for 24 hours in SHIELD-ON solution at 37 °C. All reagents were prepared using SHIELD[1] kits (LifeCanvas

Technologies, Seoul, South Korea) according to the manufacturer's instructions. For SDS-electrophoretic delipidation, SHIELD-processed brains were placed in a stochastic electrotransport machine (SmartClear Pro II, LifeCanvas Technologies, Seoul, South Korea) running at aconstant current of 1.2 A for 5-7 days.[1,2]

The whole-mount immunolabeling was performed using the modified eFLASH[2] method and SmartLabel System (LifeCanvas Technologies, South Korea). The brains were pre-incubated overnight at room temperature in sample buffer (240 mM Tris, 160 mM CAPS, 20% w/v D-sorbitol, 0.9% w/v sodium deoxycholate). Each pre-incubated specimen was placed in a sample cup (provided by the manufacturer with the SmartLabel System) containing primary, corresponding secondary antibodies and lectin diluted in 8 mL of sample buffer. Information on antibodies, lectin and their optimized quantities is detailed in supplementary table 1. The brains in the sample cup and 500 mL of labeling buffer (240 mM Tris, 160 mM CAPS, 20% w/v D-sorbitol, 0.2% w/v sodium deoxycholate) were loaded into the SmartLabel System. The device was operated at a constant voltage of 90 V with a current limit of 400 mA. After 18 hours of electrophoresis, 300 mL of booster solution (20% w/v D-sorbitol, 60 mM boric acid) was added, and electrophoresis continued for 4 hours. During the labeling, the temperature inside the device was kept at 25 °C. Labeled brains were washed twice (3 hours per wash) with PBST and then post-fixed with 4% PFA at room temperature for 1 day. Post-fixed specimens were washed twice (3 hours per wash) with PBST to remove any residual PFA. The brains were then RI-matched with NFC1 and NFC2 solutions (Nebulum technology, Taiwan) before imaging. Volumetric imaging was performed using a light-sheet microscope (SmartSPIM, LifeCanvas Technologies, South Korea) with a 3.6x customized immersion objective (NA = 0.2, working distance = 1.2 cm).[3-5]

The multiple round of immunolabeling was performed using the modified HIF-Clear protocol[3]. The fluorescence signal labeled in the previous round was bleached using a 100-W projection lamp with an LED array. After 3 days of photobleaching, specimens were incubated in 200 mM SDS at 55 °C for 1 day. The specimens were then washed in PBST to remove SDS and went for the next round of immunolabeling.

Whole brain visualization

3D visualization was performed using Imaris software (Bitplane, United Kingdom). For angiogenesis evaluation, 3 regions of interest (ROIs) close to the injury were selected (each of 0.6 × 0.6 × 0.6 mm). The surface function of Imaris software was used to segment the blood vessels and

calculate the total volume and surface area. The length and bifurcation point numbers were calculated using Vessap[4]. Erosion and dilation were performed to remove false-negative pixels and avoid false centerline detections. Next, the centerlines were extracted with a 3D thinning algorithm[5]. The bifurcation points were detected using the surrounding pixels of each point to decide a point that splits into two or more vessels. The significance of the difference in mean values was determined by means of one-way ANOVA with Tukey's multiple comparison tests at an α level = 0.05 (*$P < 0.05$; **$P < 0.01$; ***$P < 0.001$; ****$P < 0.0001$). For the GABAergic interneuron analysis shown in Figure 7b, the somatostatin-positive and calbindin positive cells were segmented using the SPOT module of Imaris based on a fluorecence intensity threshold and point spread function (PSF) size definition. For quantitation of remained wound, newborn axons, and ponto-cerebellar fiber tracts, the corresponding regions were segmented and calculated using Imaris software. The The volume of certain regions of each group was normalized by the volume of corresponding contralateral regions. All graphs were plotted using GraphPad Prism (GraphPad Software Inc., United States).

Spatial transcriptomic data processing and analysis

The 5 μm paraffin-embedded brain sections were dewaxed, hematoxylin and eosin stained, and imaged following the 10x Genomics protocol (CG000520). The Visium CytAssist library construction was followed according to the manufacturer's instructions (10x Genomics Visium CytAssit FFPE Spatial Gene Expression 11mm, Mouse). Next, the libraries were sequenced on an Illumina NovaSeq 6000 platform. After library construction and sequencing, The Tissue Microarray (TMA) 'count' method (10x Genomics SpaceRanger 2.1.1) was employed to align the probe reads to the mouse reference genome (GRCm38/mm10) using a short-read probe alignment algorithm. The resulting count matrices along with the accompanying H&E images were further analyzed using the R package Seurat (v.5.0.1) within the R environment (v.4.3.2).[6,7] First, the integration of sequencing datasets was performed through aggr pipeline settings (SpaceRanger 2.1.1). Second, the resulting gene-count matrices were normalized using SCTransform and integrated into a single object for joint processing. Third, the variable genes were projected onto a lower-dimensional space using principal component analysis (PCA) through Seurat's RunPCA function with default parameters. Last, the FindClusters function with default settings was utilized to calculate k-nearest neighbors and construct the shared nearest neighbor graph based on the Euclidean distance in the low-dimensional subspace using Seurat's FindNeighbors with dims = 1:50 and default parameters. The dimensional reduction methods, t-distributed stochastic neighbor embedding (t-SNE) and uniform manifold approximation and projection (UMAP), were performed for cell cluster visualization. For gene expression analysis, the

DEGs (p-adjust < 0.05; average $log_2FC > |0.58|$) were identified through Seurat's FindMarkers function, and the gene ontology (GO) analysis of DEGs was conducted using clusterProfiler R package (version 4.10.0).[8] Moreover, SPOTlight R package (version 1.6.7) was applied to deconvolute the spatial resolution by integrating scRNA-seq from the Mouse Cell Atlas (MCA) website (http://bis.zju.edu.cn/MCA/).[9,10] After using SPOTlight to identify cell types, subset the spots containing microglia, then input the data into the slingshot R package (version 2.10.0) to perform trajectory inference.[11]

Animal behavior testing methods

C57BL/6 female mice (7 weeks old) were randomly assigned to five experimental groups (n = 6 per group): (1) PBS control, (2) CAT@MB, (3) MB, (4) SOCO, and (5) SOCO + AMF. The cylinder test was performed following the methodology described in a previous study. In this test, forelimb asymmetry was assessed by observing spontaneous forelimb movements within a transparent cylindrical chamber (18 cm in diameter, 30 cm in height) for a 3-minute period. The chamber was designed to be sufficiently spacious to allow movement while being compact enough to encourage rearing and wall exploration. A mirror placed beneath the chamber allowed for the detailed observation and recording of forelimb movements when the mice were oriented away from direct view. Each landing event was scored based on the first limb to contact the ground or both limbs if a simultaneous touch occurred. The usage of the impaired and unimpaired forelimbs was recorded, and the limb-use asymmetry score was calculated by subtracting the percentage use of the impaired limb from that of the unimpaired limb. Additionally, wall exploration and landing behaviors were analyzed. Mice underwent testing at 7, 14, 21, 28, 35, and 42 days post-TBI following implantation.

The grid test was conducted using a wire mesh grid with square openings (2.5×2.5 cm^2) and overall dimensions of 12 cm × 36 cm × 10 cm (length × width × height). Each mouse was individually placed on the grid, and foot fault errors—defined as instances where a paw failed to land securely and slipped through the grid opening—were recorded. The foot fault rate was calculated using the formula:Foot fault rate= (Number of foot faults/Number of foot faults+Number of non-foot-fault steps)X100. Mice were tested at 7, 14, 21, 28, 35, and 42 days post-TBI following transplantation. This test provided a quantitative measure of motor coordination and limb function recovery over time.

The pasta test was used to assess fine motor control and forelimb function in mice following traumatic brain injury (TBI). Mice were food-restricted for 12 hours before testing to enhance motivation while maintaining at least 85% of their baseline body weight. Each mouse was placed in

an individual enclosure and given a standardized dry pasta stick (~5 cm in length), and their grasping ability, manipulation time, bite patterns, and total consumption time were recorded. The test evaluated forelimb coordination by analyzing limb preference and efficiency in handling the pasta, with impairments reflected in increased consumption time or altered bite patterns. Video recordings were used for detailed analysis, and testing was conducted at baseline (pre-injury) and at 7, 14, 21, 28, 35, and 42 days post-TBI. This test provided a sensitive measure of fine motor recovery and was used alongside the cylinder and grid tests for a comprehensive evaluation of functional recovery.

Data availability

The spatial transcriptomics datasets of the mouse brain for this project have been deposited in the Gene Expression Omnibus (GEO) database under accession number GSE269327.

References

1. Park, Y.-G. et al. Protection of tissue physicochemical properties using polyfunctional crosslinkers. *Nat. Biotechnol.* **37,** 73–83 (2019).
2. Yun, D. H. et al. Ultrafast immunostaining of organ-scale tissues for scalable proteomic phenotyping. *bioRxiv* 660373 (2019). https://doi.org/10.1101/660373.
3. Lin, Y.-H. et al. Revealing intact neuronal circuitry in centimeter-sized formalin-fixed paraffin-embedded brain. *Elife* **13,** (2024).
4. Todorov, M. I. et al. Machine learning analysis of whole mouse brain vasculature. *Nat. Methods* **17,** 442–449 (2020).
5. Lee, T. C., Kashyap, R. L. & Chu, C. N. Building skeleton models via 3-D medial surface axis thinning algorithms. *CVGIP Graph. Model. Image Process.* **56,** 462–478 (1994).
6. Hao, Y., Stuart, T., Kowalski, M. H. et al. Dictionary learning for integrative, multimodal and scalable single-cell analysis. *Nat. Biotechnol.* **42,** 293–304 (2024). https://doi.org/10.1038/s41587-023-01767-y.
7. R Core Team. *R: A Language and Environment for Statistical Computing*. R Foundation for Statistical Computing, Vienna, Austria (2023). https://www.R-project.org/.
8. Wu, T., Hu, E., Xu, S., Chen, M., Guo, P., Dai, Z., Feng, T., Zhou, L., Tang, W., Zhan, L., Fu, X., Liu, S., Bo, X. & Yu, G. clusterProfiler 4.0: A universal enrichment tool for interpreting omics data. *The Innovation* **2,** 100141 (2021).
9. Elosua-Bayes, M. & Crowell, H. *SPOTlight: SPOTlight: Spatial Transcriptomics Deconvolution* (2024). R package version 1.6.7. https://github.com/MarcElosua/SPOTlight.
10. Fei, L., Chen, H., Ma, L. et al. Systematic identification of cell-fate regulatory programs using a

single-cell atlas of mouse development. *Nat. Genet.* **54,** 1051–1061 (2022). https://doi.org/10.1038/s41588-022-01118-8.
11. Street, K., Risso, D., Fletcher, R. B., Das, D., Ngai, J., Yosef, N., Purdom, E. & Dudoit, S. Slingshot: cell lineage and pseudotime inference for single-cell transcriptomics. *BMC Genomics* (2018).
12.

Table S1. The staining strategies and methods for whole-brain imaging.

	Primary antibody	Secondary antibody	Lectin
Round 1	Pan-axonal filaments (SMI312) (Biolegend, 837904), 6 µg	Rhodamine red X Fab Fragment Donkey Anti-Mouse IgG (Jackson ImmunoResearch, 715-297-003), 4 µg	Lectin-Dylight 488 (Vector Labs, DL-1178-1), 40 µl
	GAD65/67 (Abcam, ab183999), 6 µg	Alexa Fluor® 647 Fab Fragment Donkey Anti-Rabbit IgG (Jackson ImmunoResearch, 711-607-003), 4 µg	
Round 2	Iba1 (Abcam, ab178847), 6 µg	Alexa Fluor® 647 Fab Fragment Donkey Anti-Rabbit IgG (Jackson ImmunoResearch, 711-607-003), 4 µg	Lectin-Dylight 488 (Vector Labs, DL-1178-1), 40 µl
	GFAP (Biolegend, 644702), 6 µg	Rhodamine red X Fab Fragment Donkey Anti-Mouse IgG (Jackson ImmunoResearch, 715-297-003), 4 µg	
Round 3	Calbindin (Cell signaling technology, 13176S), 10 ug	Alexa Fluor® 647 Fab Fragment Donkey Anti-Rabbit IgG (Jackson ImmunoResearch, 711-607-003), 6.7 µg	Lectin-Dylight 488 (Vector Labs, DL-1178-1), 40 µl
	Somatostatin (Santa Cruz, sc-47706), 10 ug	Rhodamine red X Fab Fragment Donkey Anti-Rat IgG (Jackson ImmunoResearch, 712-297-003), 6.7 µg	

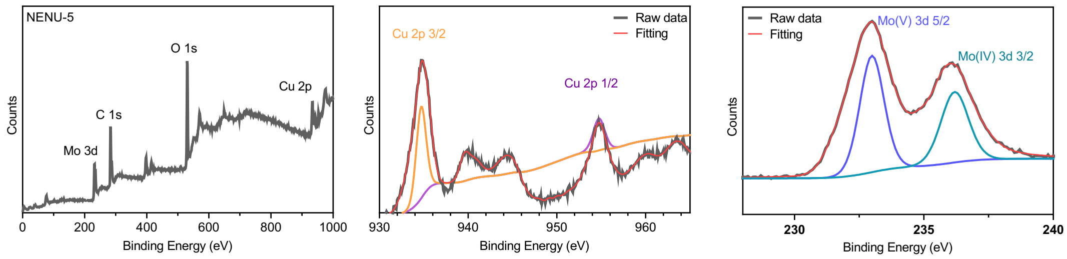

Figure S1. XPS spectra of NENU-5.

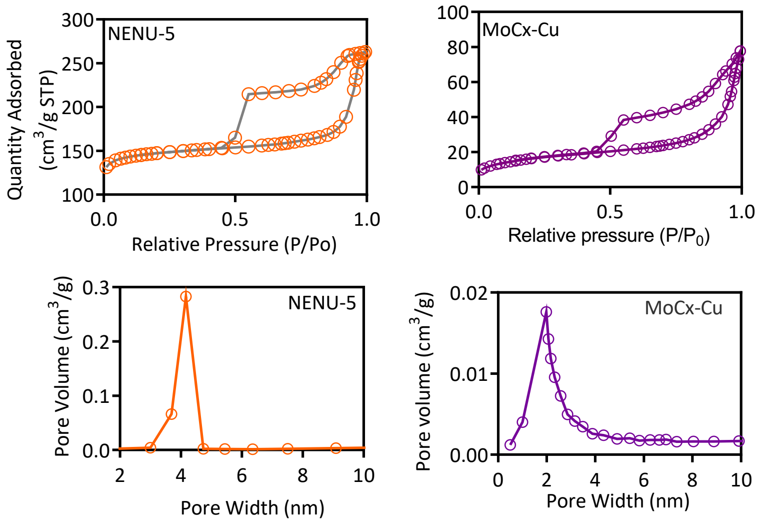

Figure S2. Brunauer–Emmett–Teller (BET) analysis of NENU-5 and MC.

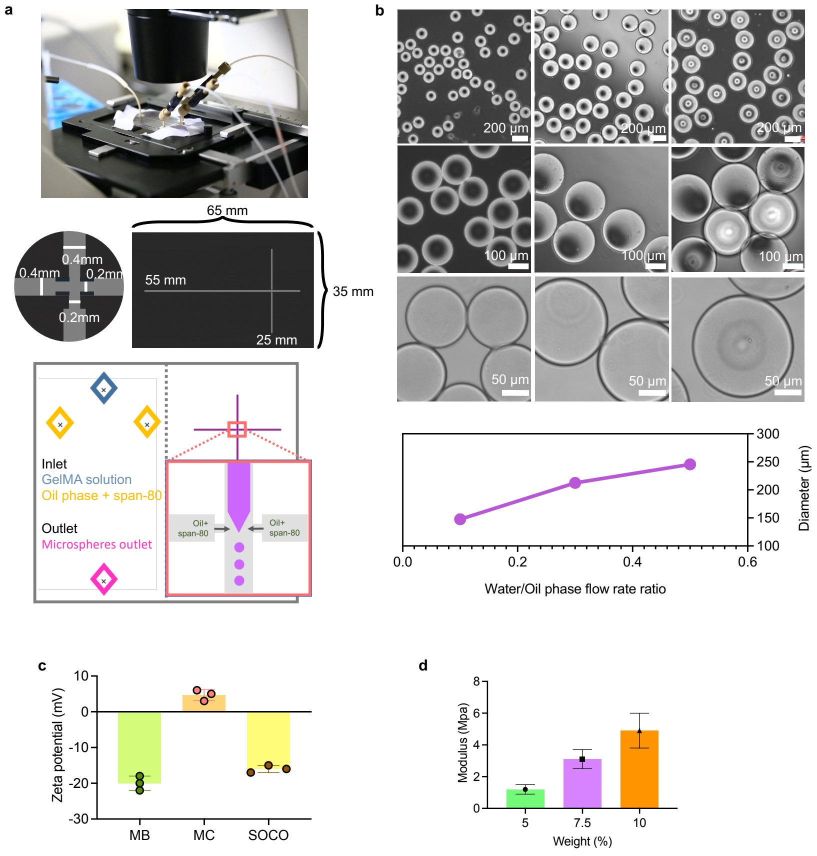

Figure S3. AutoCAD design and imaging of a microfluidic chip. (a) Photograph of the fabricated microfluidic channel. (b) Confocal and bright-field microscopy images of microspheres formed at different water/oil phase flow rates of 0.1, 0.3, and 0.5 μL/min. (c) The surface charge of MB, MC, and SOCO was determined using zeta potential measurements. Error bars represent mean ± s.d., n = 3. (d) Compressive modulus of GelMA at various concentration. Error bars represent mean ± s.d., n = 4.

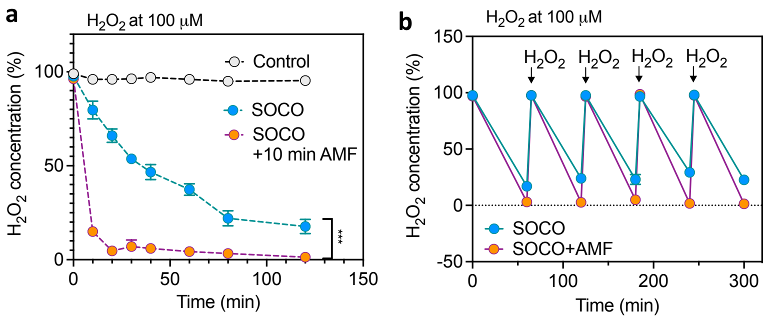

Figure S4. (a) Decomposition of H_2O_2 (100 μM) with SOCO and SOCO+AMF. (n = 5, mean ± s.d., ***$p < 0.005$, one-way ANOVA with Tukey's multiple comparison test). (c) Repetitive catalytic H_2O_2 consumption ability of SOCO and SOCO+AMF with repeated H_2O_2 additions (100 μM).

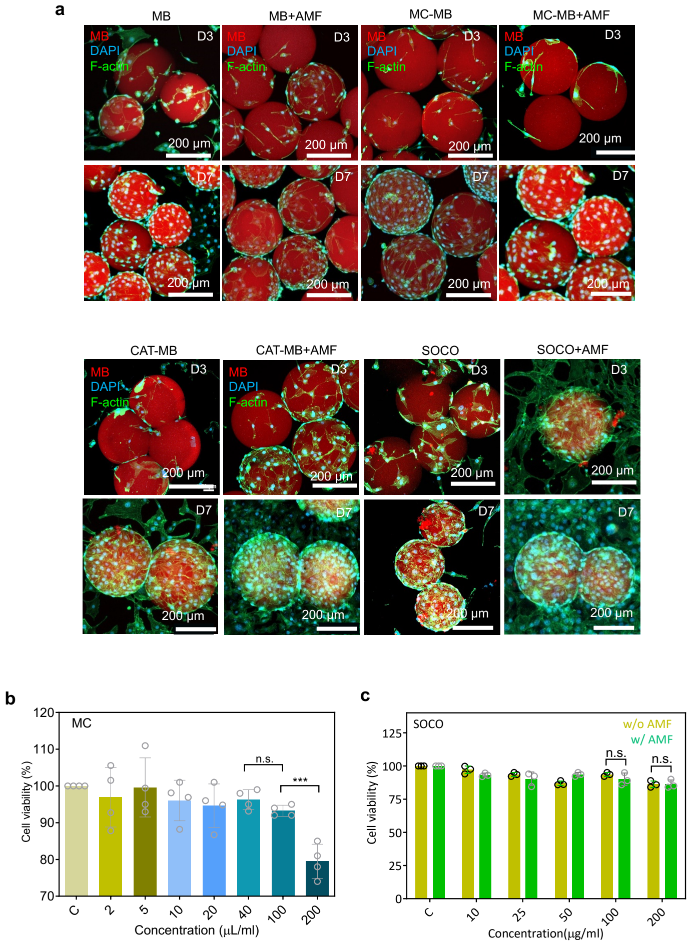

Figure S5. Multi-parametric analysis of microsphere effects on NIH-3T3 cells and neural differentiation. (a) Confocal images of various microsphere types co-cultured with NIH-3T3 cells. (b) Cell viability of NIH-3T3 cells post 24-hour incubation with supernatant from MC. (c) Cell viability after 24-hour exposure to SOCO with and without 5 min of AMF treatment. (n = 6, mean ± s.d., ***$p < 0.005$, one-way ANOVA with Tukey's multiple comparison test).

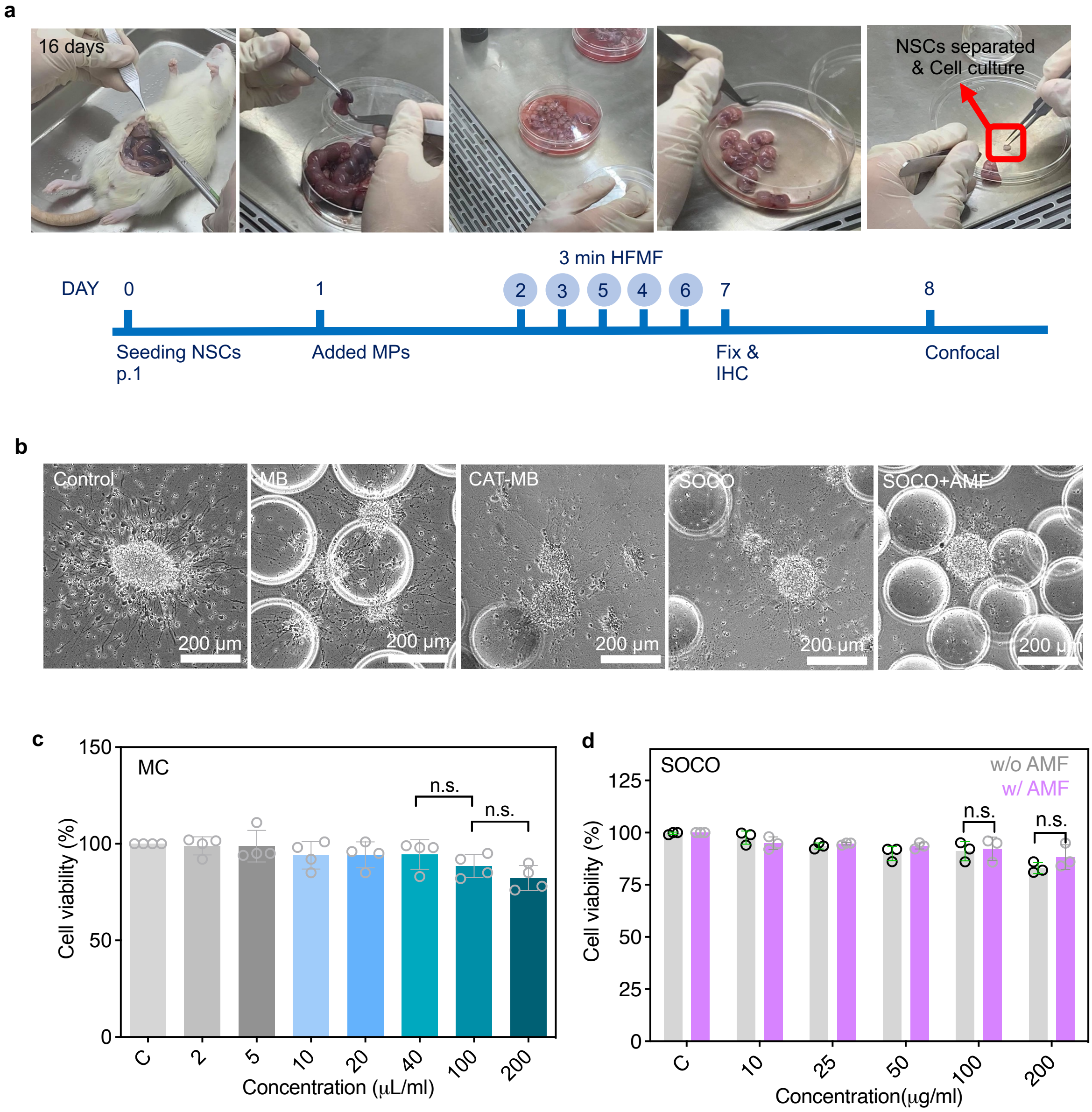

Figure S6. (a) Extraction process of primary neural stem cells. (b) Bright-field images of microspheres (MPs) co-cultured with neural stem cells (NSCs). (c) Cell viability of NSCs post 24-hour incubation with supernatant from MC. (d) Cell viability of NSCs after 24-hour exposure to SOCO with and without 5 min of AMF treatment. (n = 6, mean ± s.d., one-way ANOVA with Tukey's multiple comparison test).

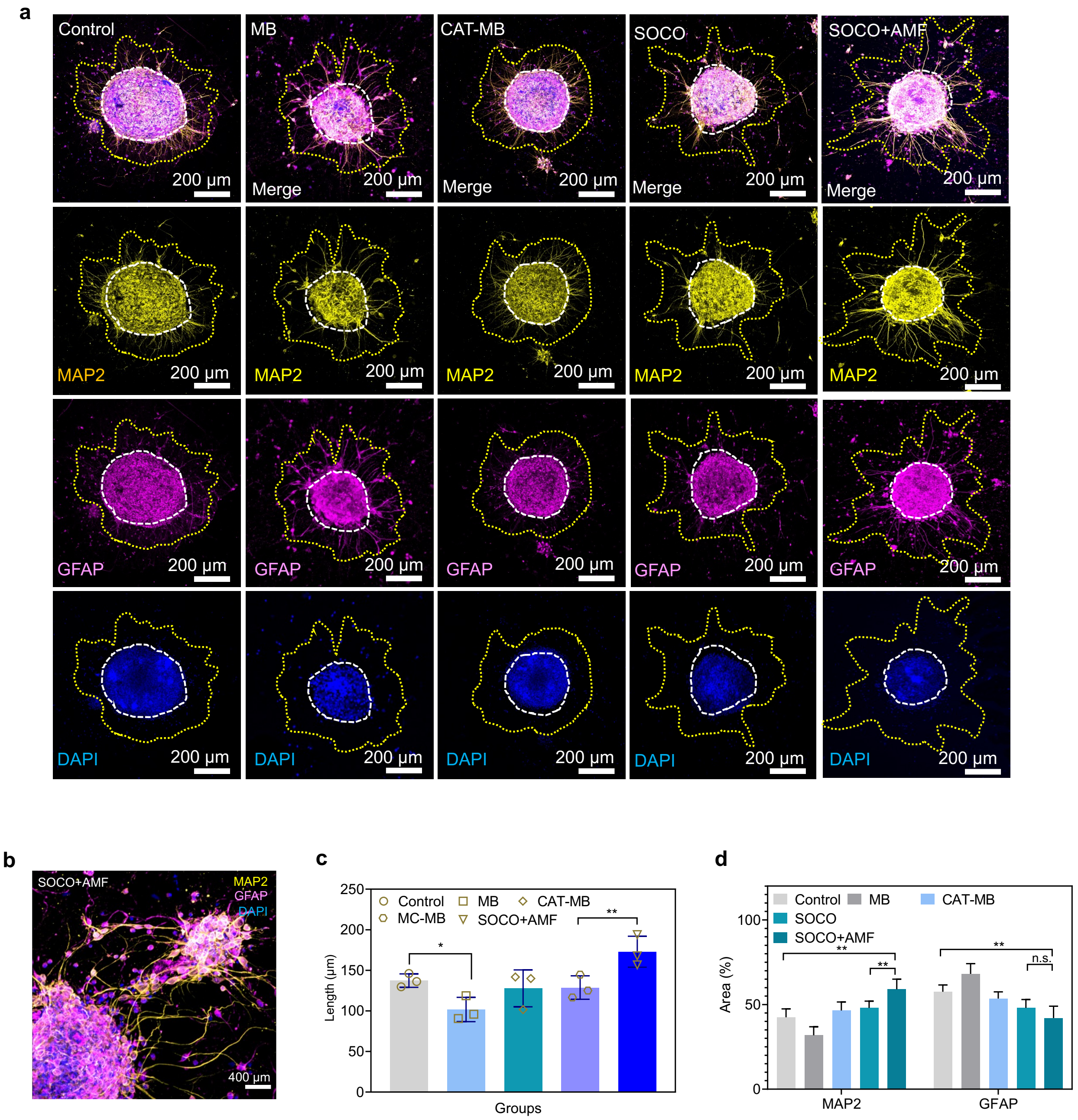

Figure S7. (a) CLSM images of NSCs treated by MB, CAT-MB, SOCO, and SOCO+AMF. In the AMF-treated group, a magnetic field with a power of 3.2 kW and a frequency of 1 MHz was applied daily for 5 minutes until the NSCs were immobilized. GFAP and MAP-2 expressions were used as markers for astrocytes and neurons, respectively. (b) CLSM images of differentiation of NSCs. (c) Quantification of axon length using ImageJ. (d) The area of MAP-2 expression after various treatments. ($n = 5$, mean ± s.d., $^{*}p < 0.05$, $^{**}p < 0.01$, one-way ANOVA with Tukey's multiple comparison test).

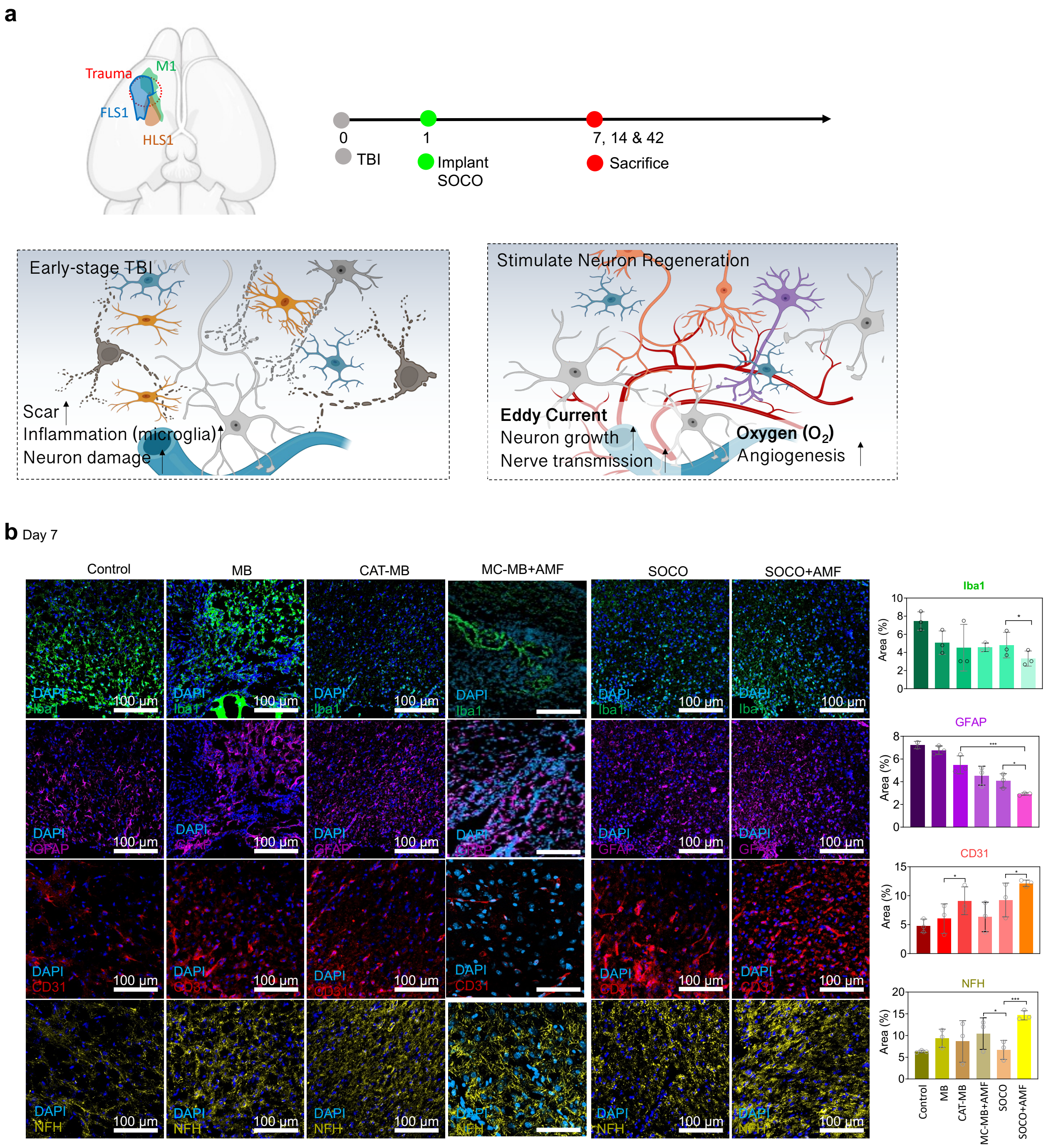

Figure S8. (a) Animal study of traumatic brain injury (TBI) and treatment schedules. Illustration of TBI and experimental timeline for treatment and sacrifice/analysis. Short- and long-term stages of repair. (b) CLSM images of areas adjacent to the injury site, showing astrocytes (purple, stained with GFAP), microglia/macrophages (green, stained with Iba1), neurofilament cells (yellow, stained with NF200), and blood vessels (red, stained with CD31) at 7 post-injury. Blue fluorescence represents nuclei stained with DAPI (n = 5, mean ± s.d., *p < 0.05, **p < 0.01, one-way ANOVA with Tukey's multiple comparison test).

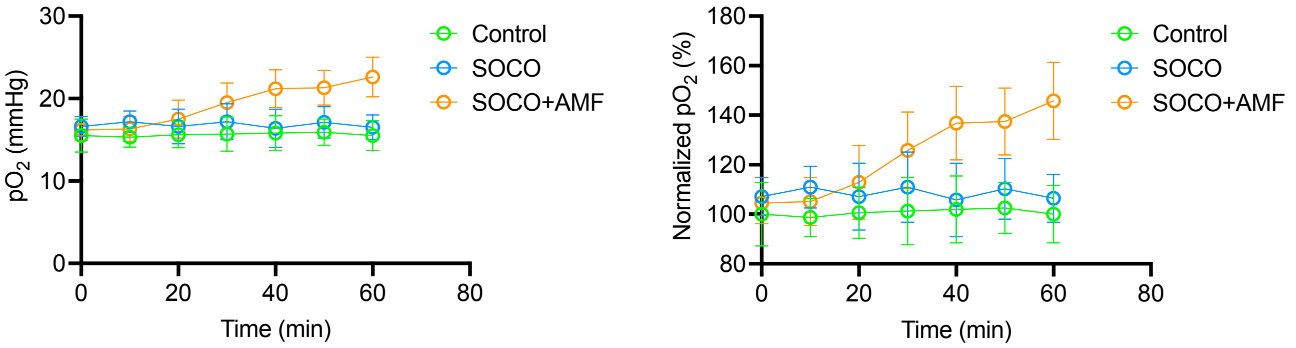

Figure S9. The pO_2 levels at TBI at 0 to 60 min in the control, SOCO and SOCO+AMF groups, respectively.

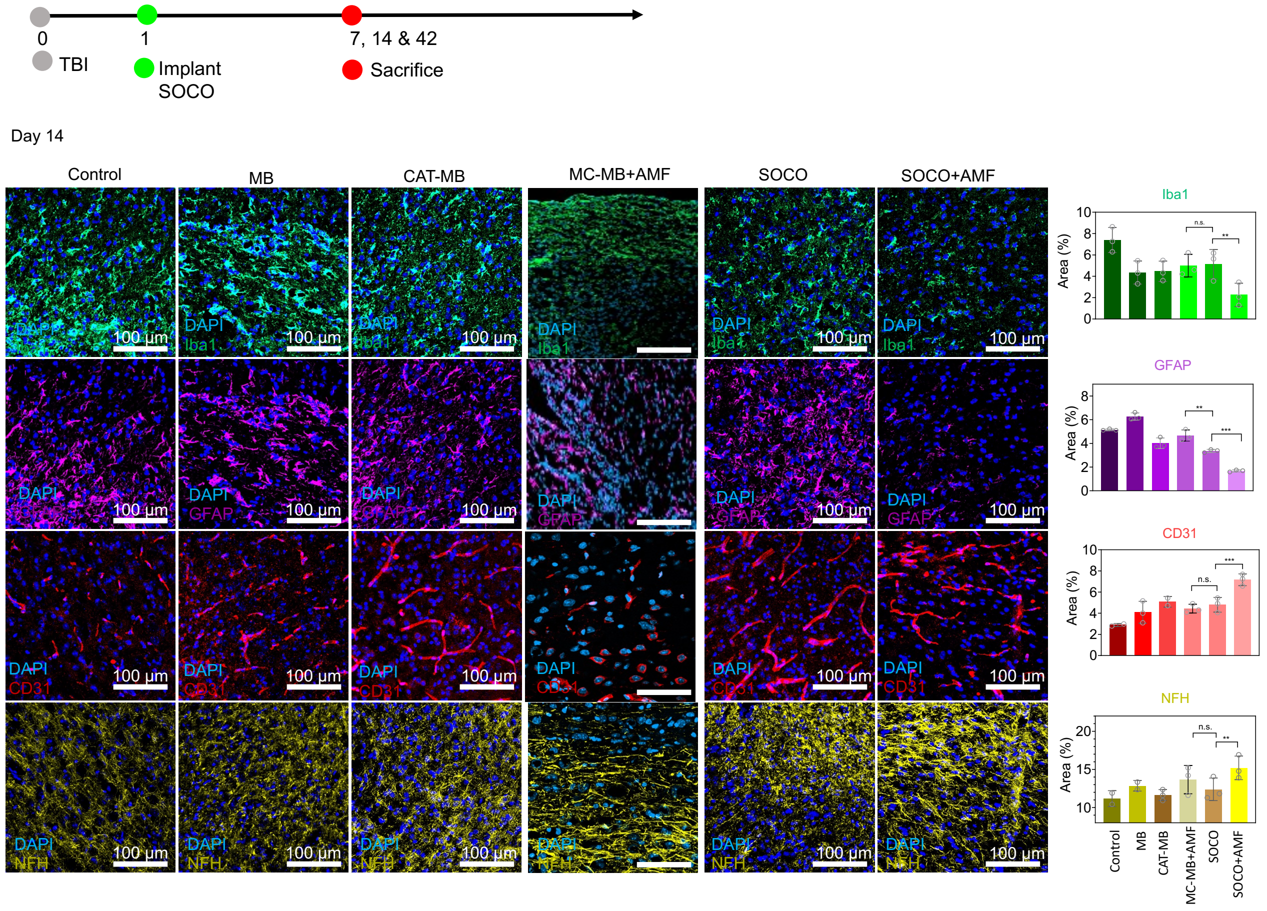

Figure S10. CLSM images of areas adjacent to the injury site, showing astrocytes (purple, stained with GFAP), microglia/macrophages (green, stained with Iba1), neurofilament cells (yellow, stained with NF200), and blood vessels (red, stained with CD31) at 14 post-injury. Blue fluorescence represents nuclei stained with DAPI (n = 5, mean ± s.d., *p < 0.05, **p < 0.01, one-way ANOVA with Tukey's multiple comparison test).

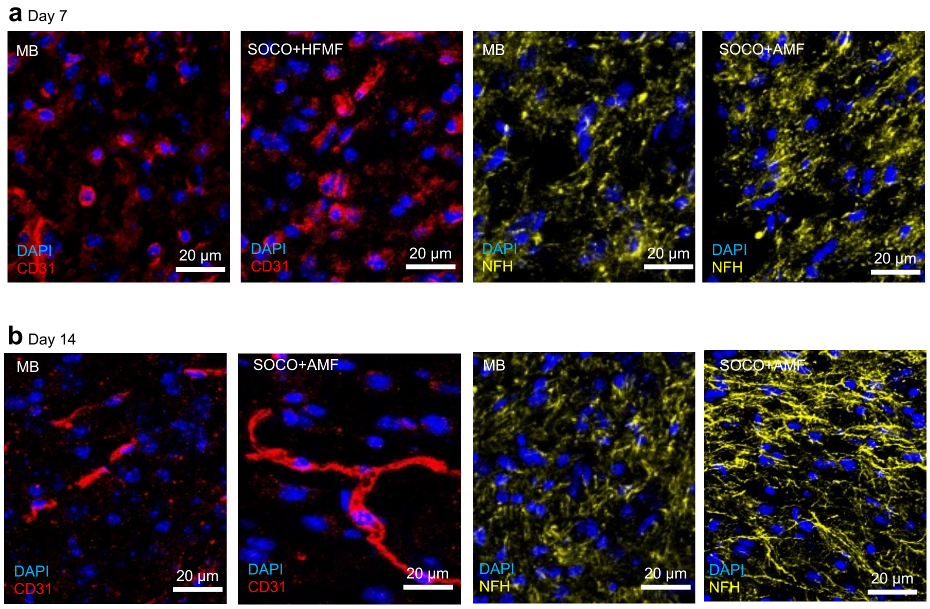

Figure S11. CLSM images of areas adjacent to the injury site, showing blood vessels (red, stained with CD31) and neurofilament cells (yellow, stained with NF200) at (a) 7 and (b) 14 days post-injury.

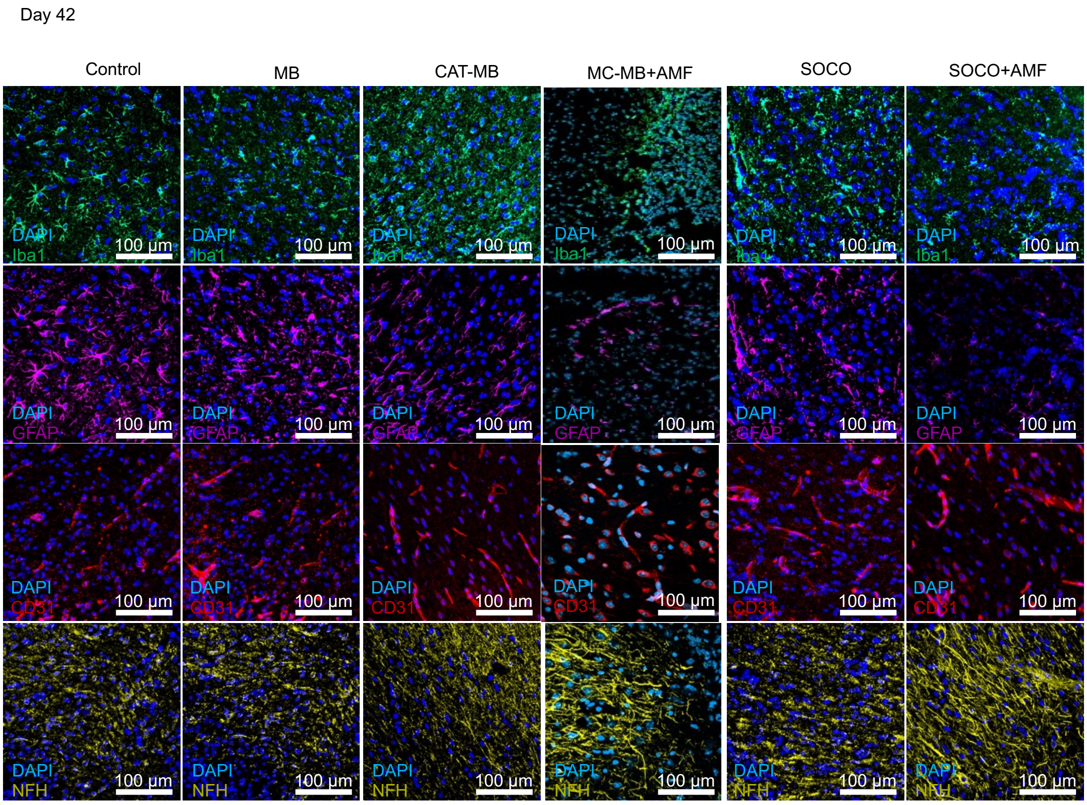

Figure S12. CLSM images of areas adjacent to the injury site, showing astrocytes (purple, stained with GFAP), microglia/macrophages (green, stained with Iba1), neurofilament cells (yellow, stained with NF200), and blood vessels (red, stained with CD31) at 42 post-injury.

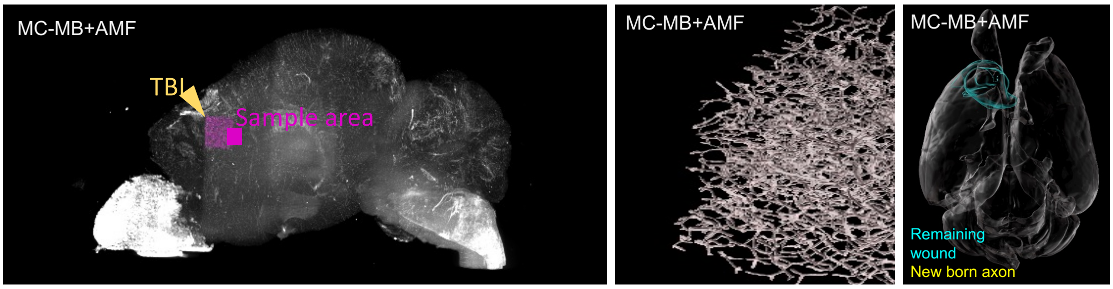

Figure S13. Reconstructed 3D images of blood vessels near the injury site after treated by MC-MB+AMF.

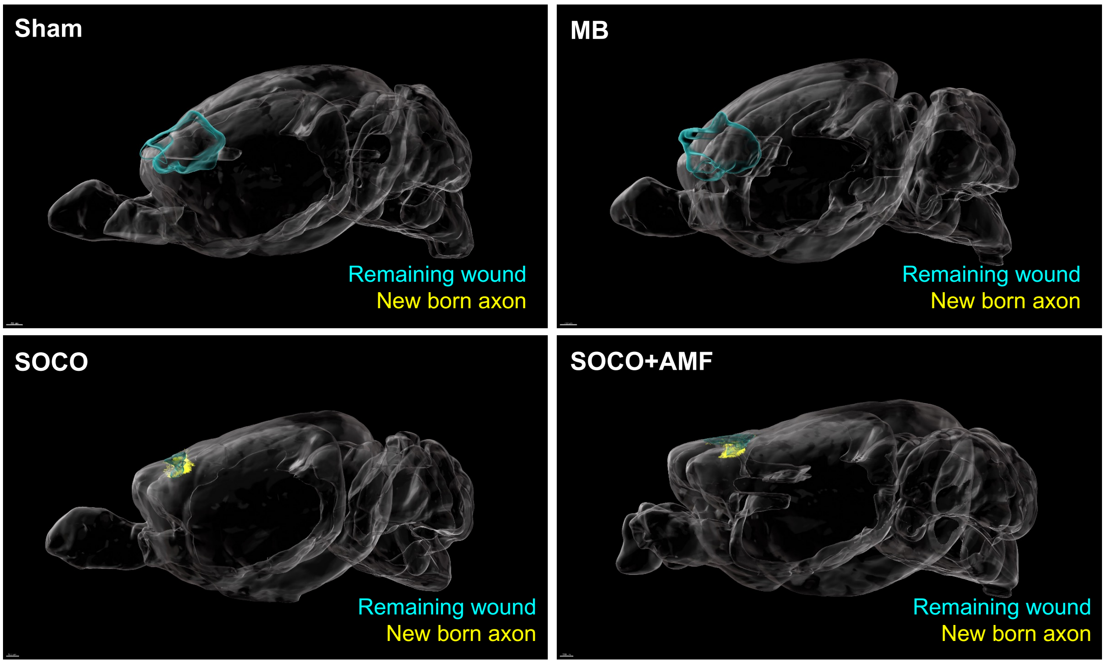

Figure S14. Reconstructed 3D images of newborn neurons at TBI site.

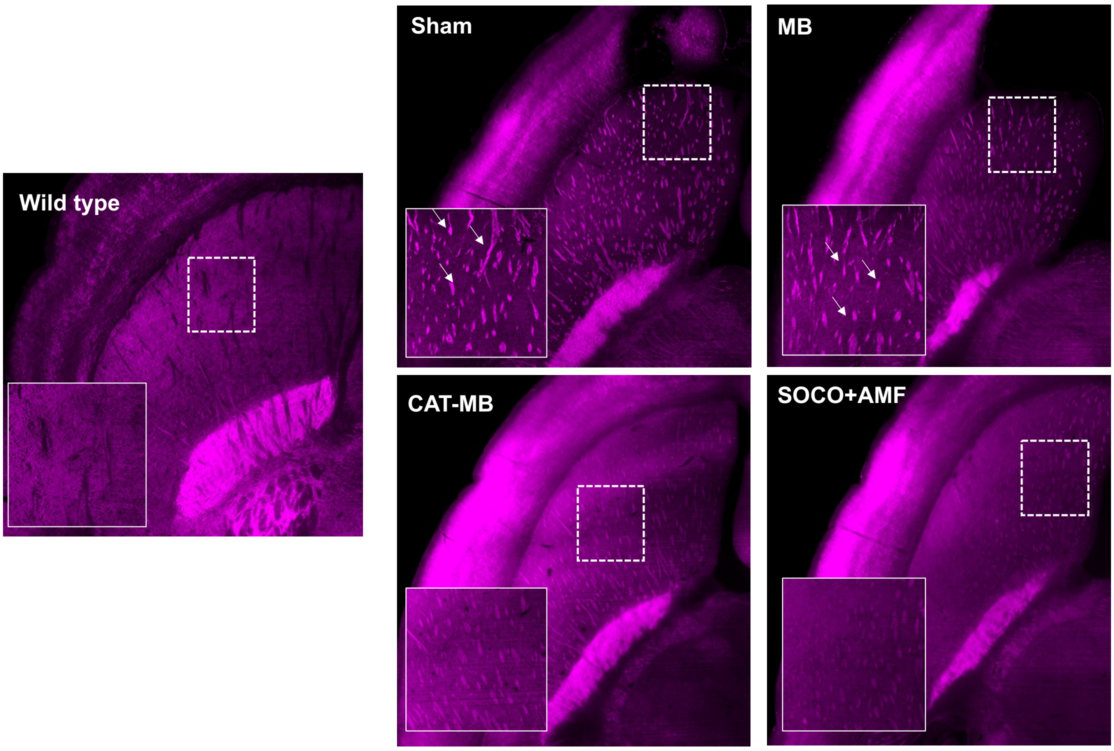

Figure S15. Reconstructed 3D images of GABAergic expression in the whole brain and at the injury site.

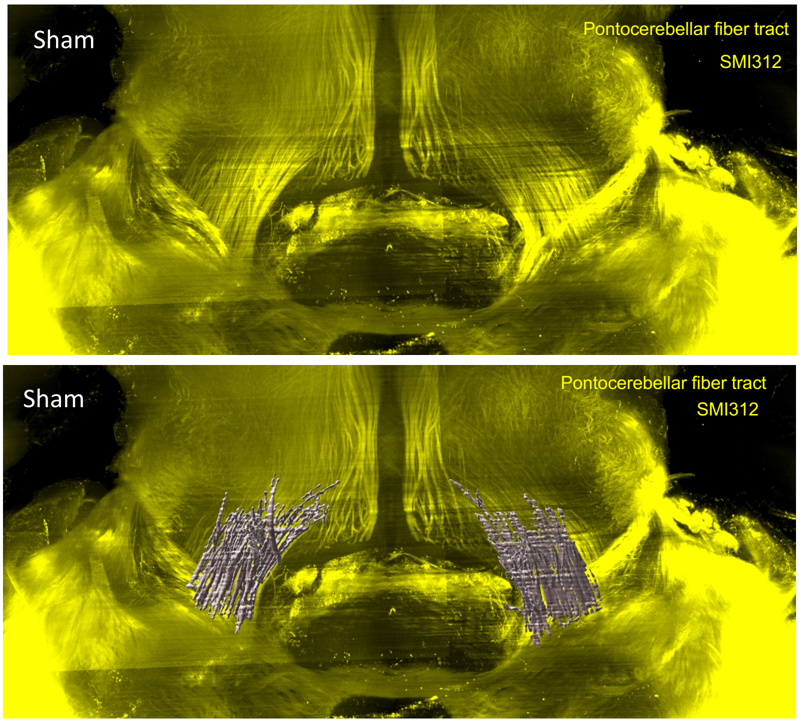

Figure S16. Reconstructed 3D images of pontocerebellar fiber tract after various treatment.

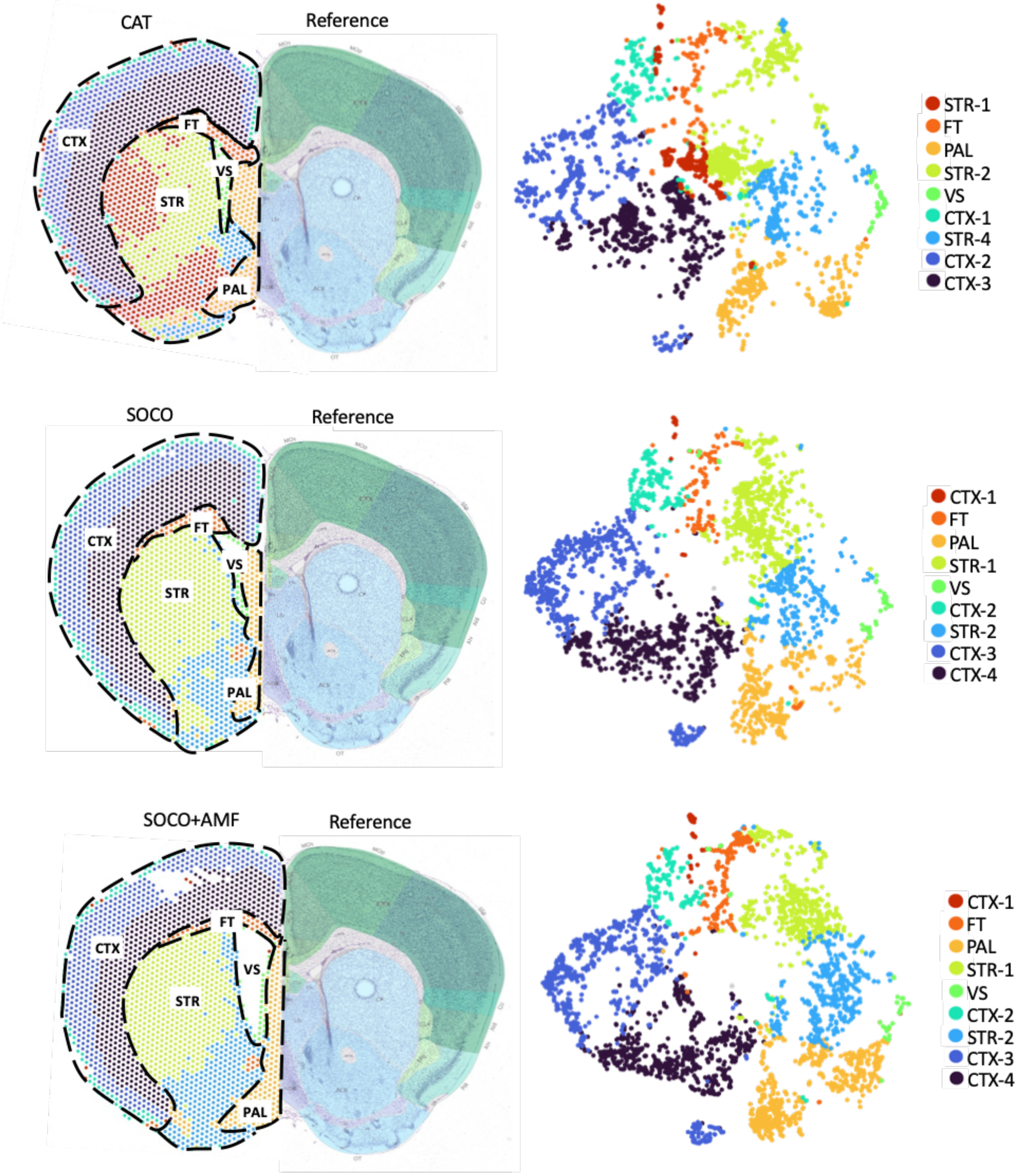

Figure S17. Nine identical brain regions were categorized into CAT, SOCO, and SOCO+AMF groups.

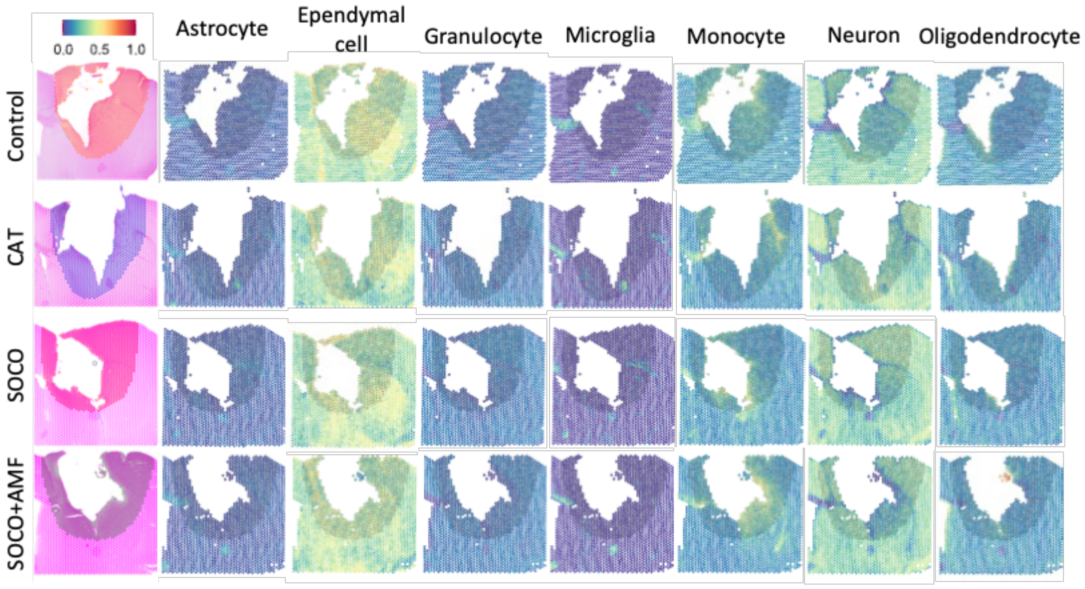

Figure S18. The changes in cell type composition in response to nanoparticle treatment, specifically in the areas surrounding the injury site.

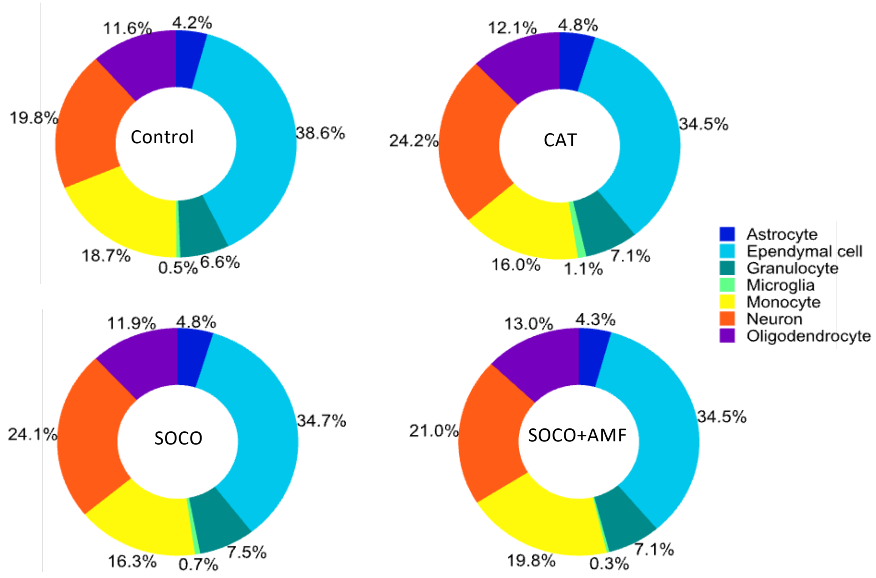

Figure S19. The percentage of identified cell types surrounding the injury site after various treatments.

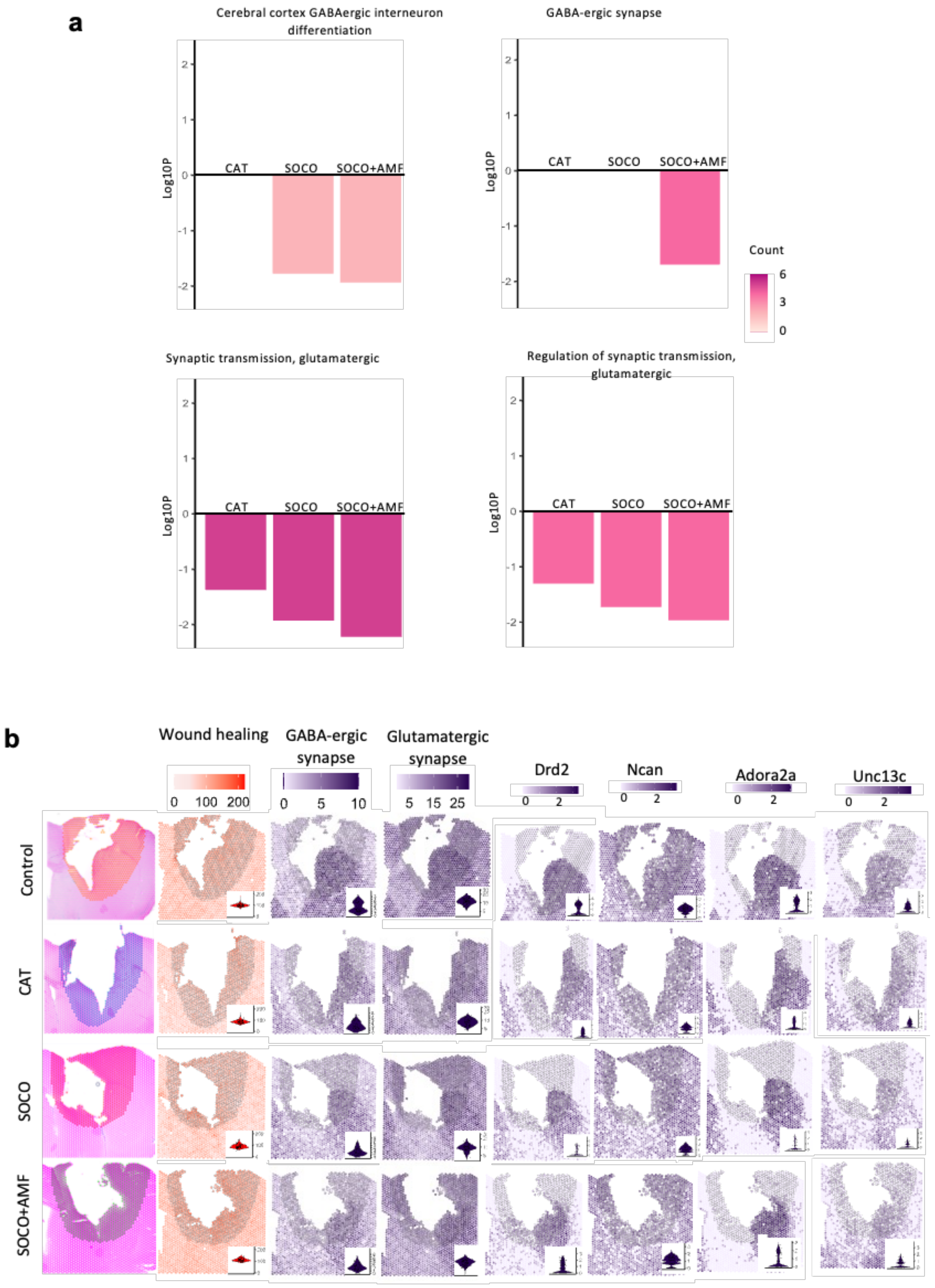

Figure S20. (a) Bat plot of enriched biological processes associated with synaptic transmission after various treatments. Color indicates gene count. (b) Spatial gene expression maps for neurontransmitter transmission and wound-related genes and GO terms across various brain injury regions, visualized through sequential brain sections.
